# Supplementary figures and images for: Call it a conspiracy: How conspiracy belief predicts recognition of conspiracy theories
Source: PLoS One. 2024 Apr 18;19(4):e0301601. doi: 10.1371/journal.pone.0301601 (PMC11025851; doi:10.1371/journal.pone.0301601)

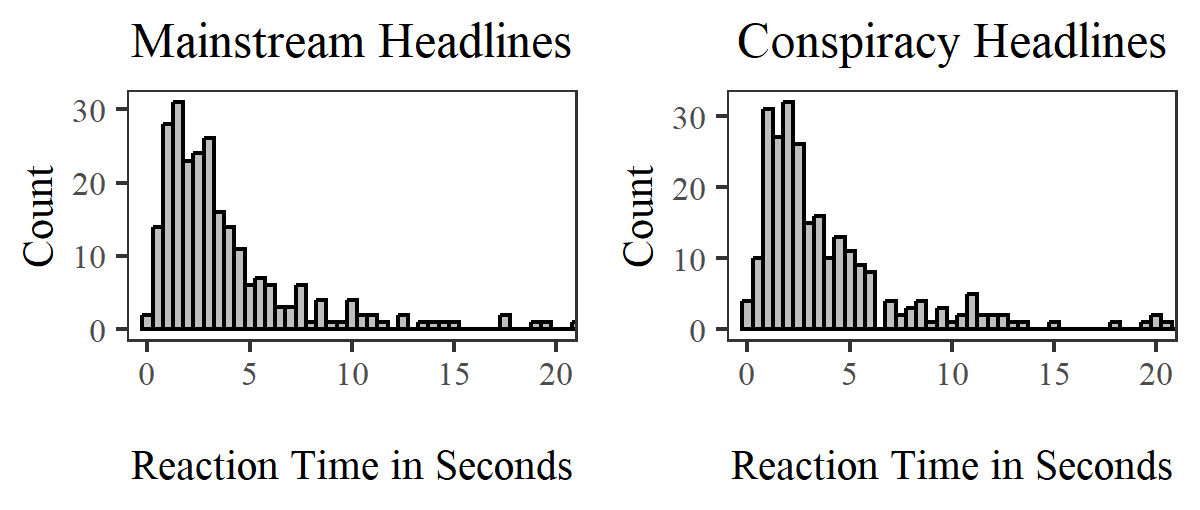

Supplement: S1 Fig — (TIF) [file pone.0301601.s001.tif]

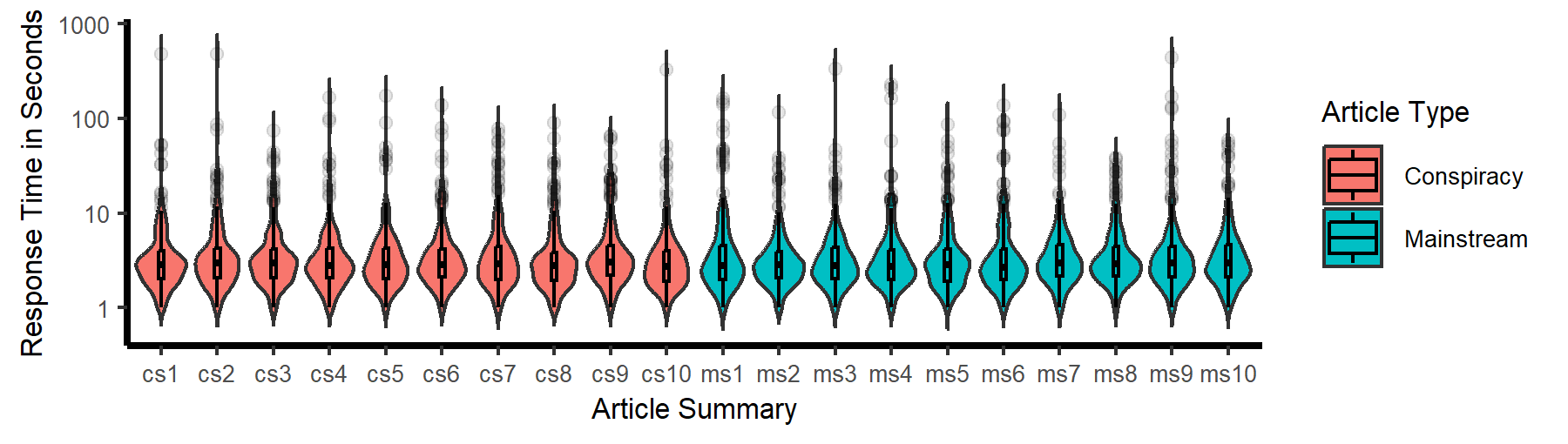

Supplement: S2 Fig — (TIF) [file pone.0301601.s002.tif]

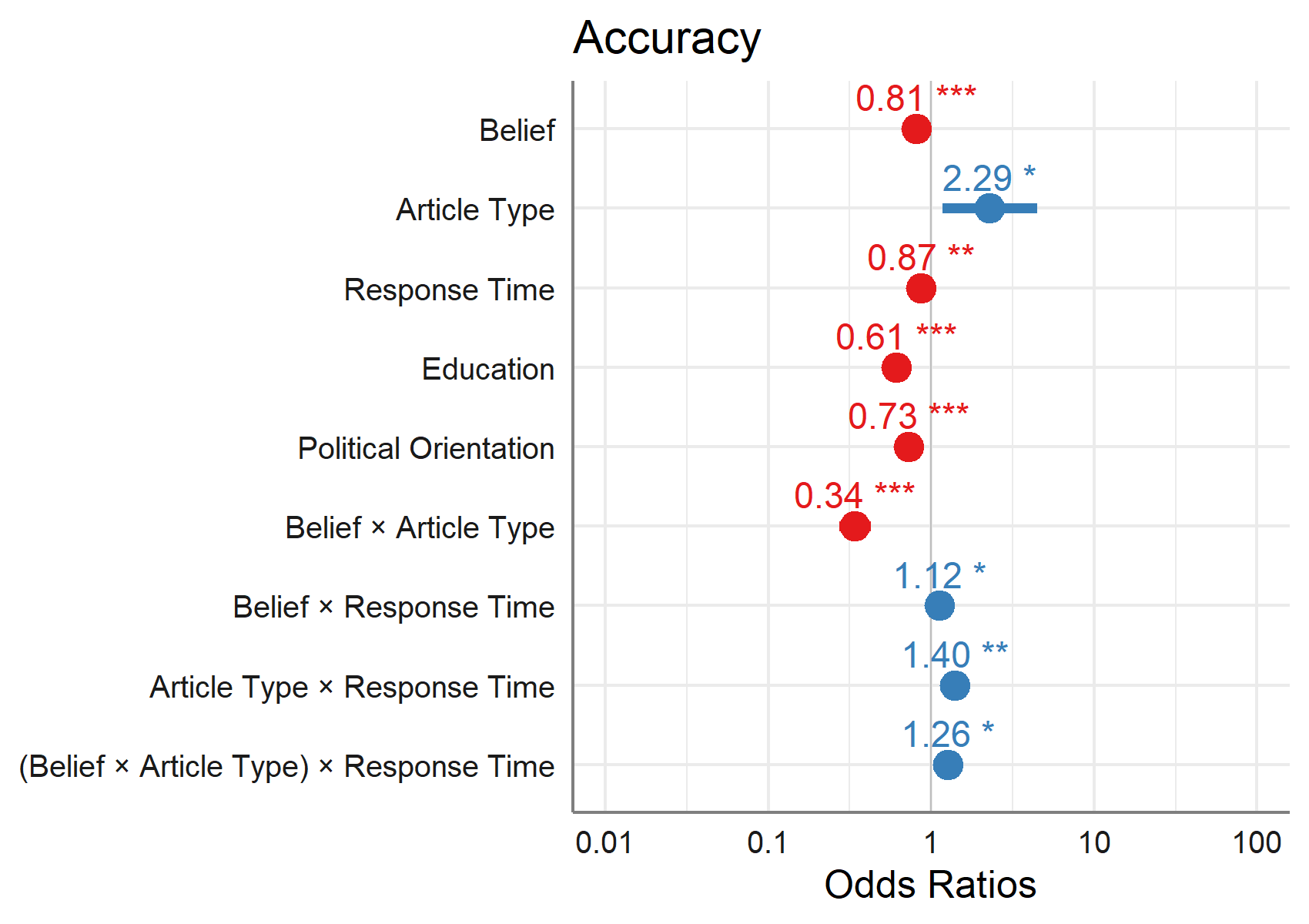

Supplement: S3 Fig — (TIF) [file pone.0301601.s003.tif]

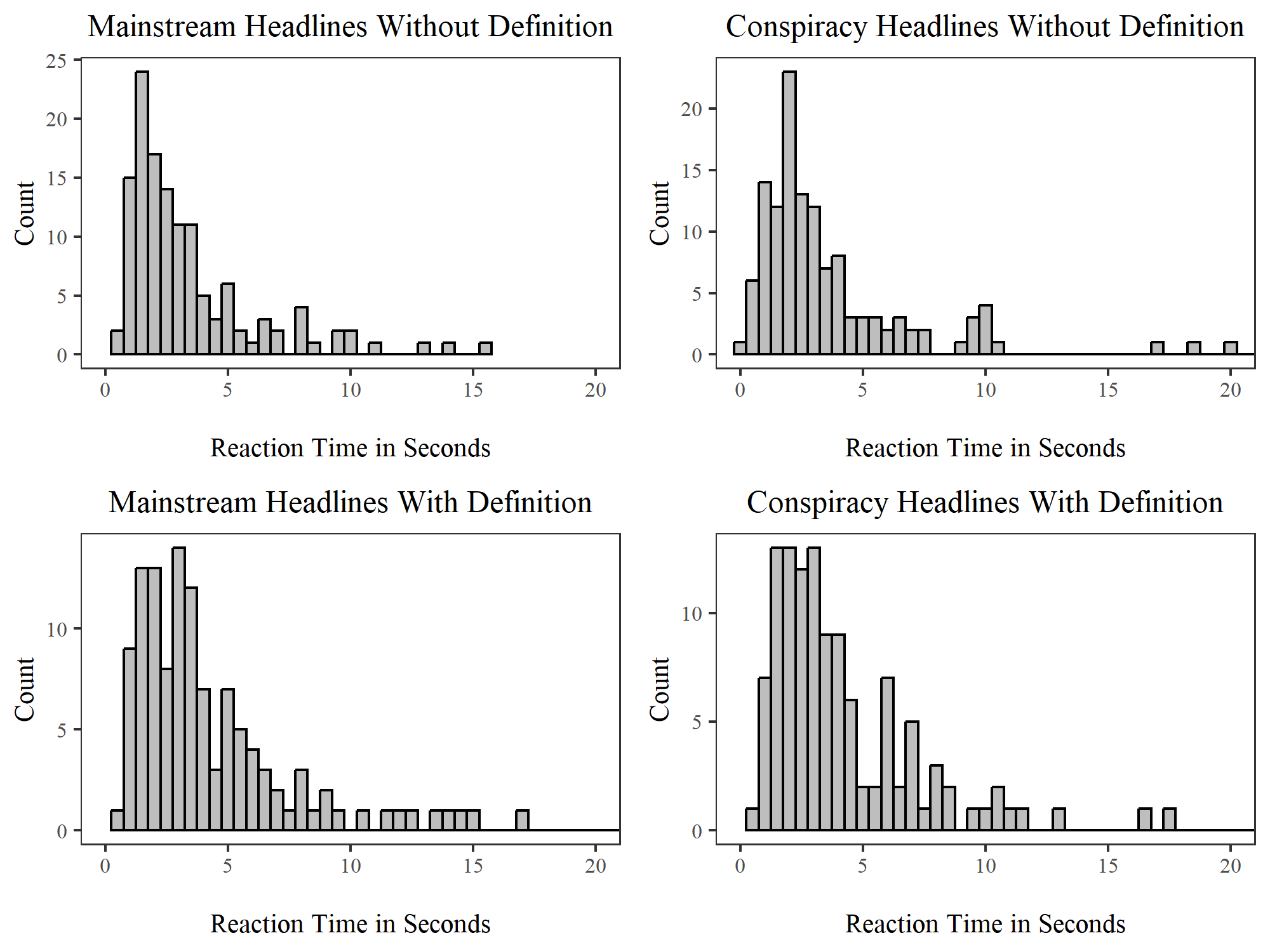

Supplement: S4 Fig — (TIF) [file pone.0301601.s004.tif]

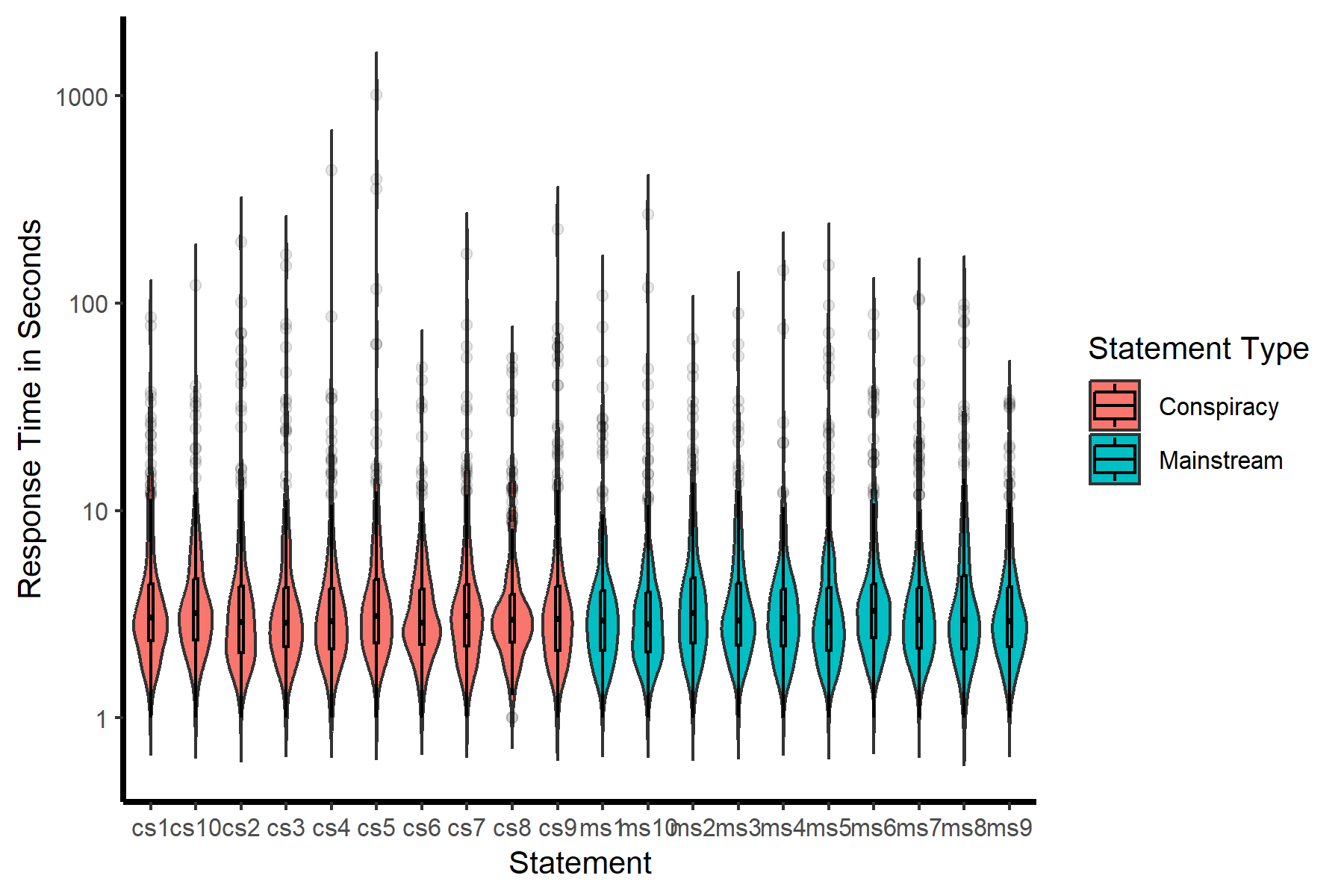

Supplement: S5 Fig — (TIF) [file pone.0301601.s005.tif]

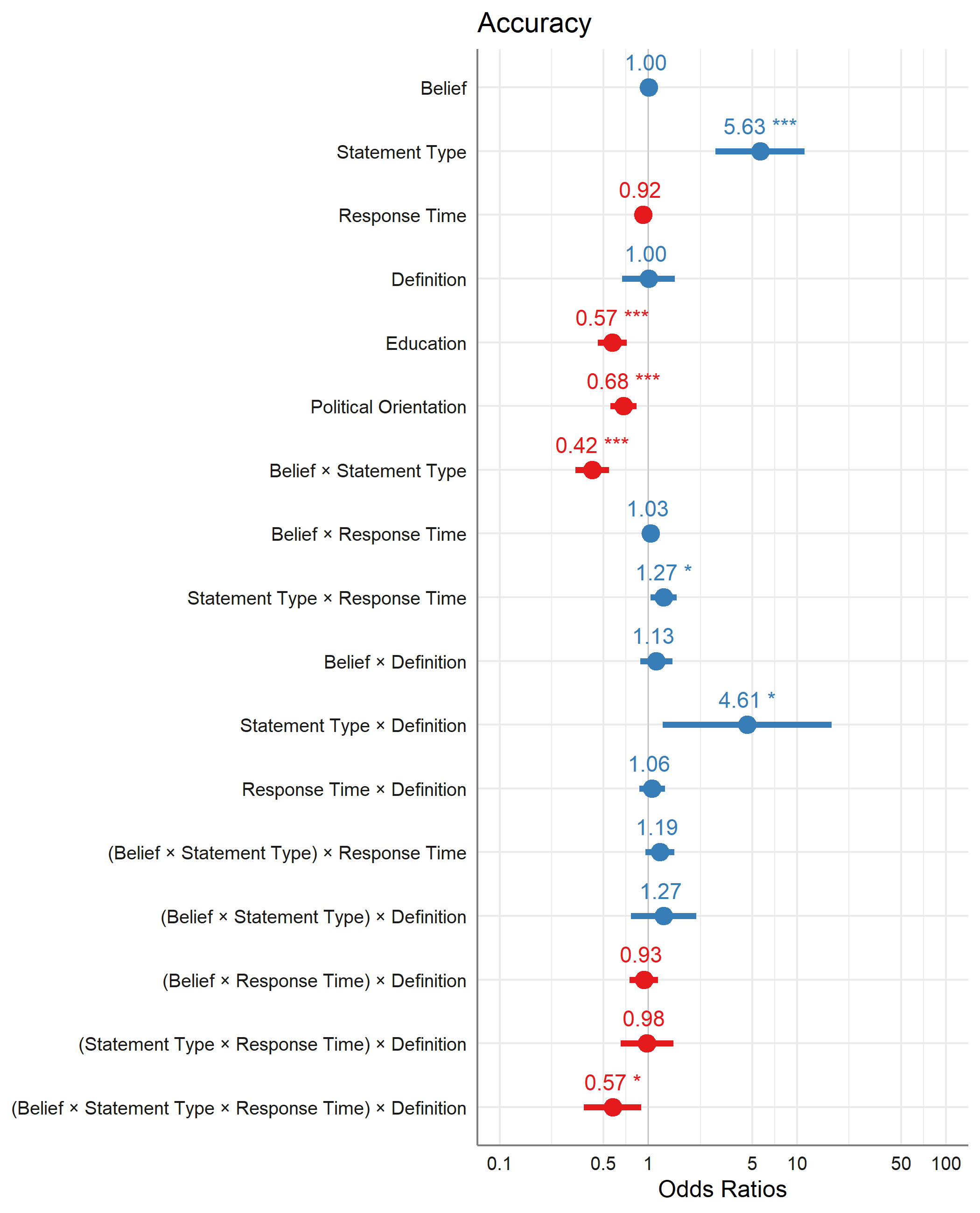

Supplement: S6 Fig — (TIF) [file pone.0301601.s006.tif]
