# Supplementary material for: Call it a conspiracy: How conspiracy belief predicts recognition of conspiracy theories
Source: PLoS One. 2024 Apr 18;19(4):e0301601. doi: 10.1371/journal.pone.0301601 (PMC11025851; doi:10.1371/journal.pone.0301601)
Supplement: S1 Table — (DOCX) [file pone.0301601.s007.docx]

*S1 Table.* Means and standard deviations of belief, the proportion of participants who saw all three conspiracy features, correlations between political orientation and belief, and interrater agreement on the presence of all three conspiracy features

| Statement | Belief  *M* (*SD*) | Proportion | *r* | Agreement |
| --- | --- | --- | --- | --- |
| **Conspiracy Statements** |  |  |  |  |
| *COVID-19: Perfect Cover for Mandatory Biometric ID* | 0.13 (1.97) | .64 | -.38*** | Yes |
| CNN heavily revises article called out for looking suspiciously like Chinese news release | 0.25 (1.69) | .49 | -.36*** | Yes |
| *Coming to a wall or lamppost near you – 5G and fake diseases to cover up its effects* | -0.33 (2.01) | .58 | -.29** | Yes |
| *New WikiLeaks Documents Expose Doctoring of Chemical Weapons Report to Justify 2018 US Attack on Syria* | 0.03 (1.56) | .58 | -.33** | Yes |
| *Psychologist: big tech will use “subliminal methods” to shift 15 million votes on election day* | -0.25 (1.90) | .67 | -.32** | Yes |
| China launches biological warfare agenda: Covertly infiltrates plane with “Trojan Horse” coronavirus carrier | -0.56 (2.03) | .63 | -.43*** | Yes |
| Cover-up: Iran refuses to hand over black box data of plane that crashed over Tehran | 0.79 (1.48) | .45 | -.11 | No |
| *“Undeniable evidence”: Explosive classified docs reveal Afghan war mass deception* | 0.57 (1.63) | .62 | -.13 | Yes |
| The same people who lied about WMDs in Iraq are pushing conflict with Iran | 0.52 (1.52) | .50 | -.07 | Yes |
| *Alarming report reveals secretive surveillance state powered by your phone’s location services* | 0.67 (1.65) | .61 | -.27* | Yes |
| *New “Out of Shadows” documentary exposes the media and Hollywood for manipulating the masses with lies and propaganda* | 0.39 (1.76) | .53 | -.33** | Yes |
| Ads warning about dangers of 5G banned by Great Britain’s advertising “authority” | 0.27 (1.81) | .43 | -.31** | No |
| What Happened on the Planes on September 11, 2001? The 9/11 Cell Phone Calls. The 9/11 Commission “Script” Was Fabricated | -0.29 (1.94) | .41 | -.38*** | Yes |
| *US Congress cracks down on ABC News for ‘Epstein coverup,’ demands to know who killed the story and why* | 0.80 (1.41) | .50 | -.28** | Yes |
| *Did someone murder the wife of a Google whistleblower whose research implicated the tech giant in election meddling?* | -0.31 (1.97) | .55 | -.30** | Yes |
| **Non-Conspiracy Statements** |  |  |  |  |
| *WHO warning: No evidence that antibody tests can show coronavirus immunity* | 1.13 (1.53) | .20 | .21 | Yes |
| Pentagon to extend troop movement freeze to June 30 | 1.27 (1.41) | .22 | -.04 | Yes |
| American voters worry they can’t spot misleading information, poll finds | 1.40 (1.39) | .30 | .16 | Yes |
| Iranian Missile Accidentally Brought Down Ukrainian Jet, Officials Say | 0.90 (1.52) | .28 | -.03 | Yes |
| *New Google site shows where people in a community are taking social distancing seriously — and where they're not* | 1.44 (1.30) | .21 | .07 | Yes |
| *FDA approves new drug for patients with metastatic breast cancer* | 1.38 (1.16) | .18 | -.02 | Yes |
| *Canada shooting: gunman kills 16 people after rampage in Nova Scotia* | 1.32 (1.47) | .20 | .24* | Yes |
| *Appeals court sides with feds on Jeffrey Epstein deal* | 0.65 (1.46) | .20 | -.10 | Yes |
| *World News Updates: Singapore’s Control Slips, as Europe Begins to Ease Coronavirus Limits* | 1.30 (1.31) | .18 | .20 | Yes |
| *East Bay student who made ‘terrorists’ video settles with school district over free speech lawsuit* | 0.63 (1.53) | .20 | -.14 | Yes |
| *DOJ review finds material errors in two 2019 surveillance applications* | 1.07 (1.18) | .21 | .08 | Yes |
| Iran president says Iran responded, will respond to assassination of Soleimani | 1.00 (1.61) | .24 | -.02 | Yes |
| Wikileaks founder Julian Assange denied bail by London court | 1.11 (1.50) | .22 | .01 | Yes |
| *New York 9/11 victim identified 18 years later* | 1.04 (1.59) | .21 | .03 | Yes |
| *Afghan conflict: US and Taliban sign deal to end 18-year war* | 1.12 (1.38) | .19 | -.15 | Yes |

*Note.* Headlines selected for the final study are in italics.
