# Supplementary material for: Call it a conspiracy: How conspiracy belief predicts recognition of conspiracy theories
Source: PLoS One. 2024 Apr 18;19(4):e0301601. doi: 10.1371/journal.pone.0301601 (PMC11025851; doi:10.1371/journal.pone.0301601)
Supplement: S2 Table — (DOCX) [file pone.0301601.s008.docx]

*S2 Table.* Means and standard deviations of belief, the proportion of participants who saw all three conspiracy features, correlations between political orientation and belief, and interrater agreement on the presence of all three conspiracy features

| Statement | Belief  *M* (*SD*) | Proportion | *r* | Agreement |
| --- | --- | --- | --- | --- |
| **Conspiracy Statements** |  |  |  |  |
| Lobbyists for pharmaceutical companies are pushing state governments to require vaccinations. | 0.40 (1.81) | .52 | -.39*** | Yes |
| The Chinese government is using its influence to force Google to suppress unfavorable information. | 0.92 (1.70) | .52 | -.06 | Yes |
| *Companies that sell smart technology like Google Home and Alexa are collecting information on their customers without their customers’ knowledge and selling that information to third parties.* | 0.77 (1.59) | .62 | -.11 | Yes |
| *COVID-19 (“the coronavirus”) was created in a lab in China as a bioweapon.* | -0.61 (2.16) | .62 | -.51*** | Yes |
| *Technology companies are suppressing information on the negative health effects of 5G networks.* | -0.39 (1.96) | .64 | -.44*** | Yes |
| *Several members of UK's Parliament were behind the 2005 London bombings in an attempt to increase support for military intervention in the Middle East.* | -0.11 (1.98) | .63 | -.34** | Yes |
| Amazon's publicity department has been paying television stations to air scripted statements disguised as news reports. | 0.30 (1.65) | .45 | -.15 | Yes |
| *Researchers have discovered a cure for cancer, but pharmaceutical companies are suppressing information about it.* | -0.15 (2.08) | .59 | -.39*** | Yes |
| *Jeffery Epstein was assassinated to prevent him from sharing information that would harm powerful politicians.* | 0.57 (1.59) | .59 | -.28* | No |
| *The New England Patriots won against the Jacksonville Jaguars in the 2018 NFL Playoffs because they’d paid off the referees to make calls in their favor.* | -0.12 (1.85) | .65 | -.38*** | Yes |
| *The U.S. government faked the moon landing to gain an advantage in the Cold War over Russia.* | -0.37 (2.20) | .54 | -.42*** | Yes |
| Paul McCartney died in the 1960s, and his music label replaced him with a look-alike to avoid losing money | -0.86 (2.02) | .40 | -.41*** | Yes |
| *Princess Diana was assassinated to prevent her from embarrassing the royal family.* | -0.33 (2.01) | .54 | -.36*** | Yes |
| There is a secret weapons testing facility hidden under the Denver Airport. | -0.25 (1.94) | .35 | -.36*** | Yes |
| *During the Cold War, the KGB assassinated several scientists that were working on US defense department projects.* | 0.72 (1.44) | .59 | -.22* | Yes |
| **Non-Conspiracy Statements** |  |  |  |  |
| Several army veterans bombed a federal building in Oklahoma City as retaliation for federal government’s perceived incompetence in several investigations. | 0.69 (1.78) | .29 | -.02 | No |
| The U.S. sent troops to Libya to assist its government in its conflict with several militant groups. | 1.08 (1.20) | .21 | -.18 | Yes |
| *Tech companies are investing in new technology that will allow them to automate various tasks including checking out customers at stores and packaging products for shipment.* | 1.56 (1.17) | .18 | .14 | Yes |
| Some governments are tracking the movement of people who were later diagnosed with COVID-19 to predict which communities will need the most resources. | 1.41 (1.28) | .21 | .12 | Yes |
| *All 50 states in the U.S. require that students are vaccinated before enrolling in public schools, though some exemptions are available for health and religious reasons.* | 1.43 (1.38) | .19 | .11 | Yes |
| *The man who drove a car into counter-protesters during the “Unite the Right” rally in Charlottesville, Virginia was charged with first-degree murder and various other offenses.* | 1.52 (1.40) | .18 | .29** | Yes |
| *The International Monetary Fund is an international organization that encourages economic cooperation and provides loans to countries in need.* | 1.39 (1.37) | .18 | .10 | Yes |
| *Scientists are developing a method to create 3-D printed organs for patients in need.* | 1.22 (1.40) | .15 | -.02 | Yes |
| *John Lennon was murdered by a man who wanted media attention.* | 1.54 (1.54) | .17 | .09 | Yes |
| *The Toronto Raptors won against the Golden State Warriors in the 2019 NBA finals, winning four of the six games in the series.* | 1.44 (1.57) | .18 | .04 | Yes |
| Martin Luther King Jr. was assassinated by an escaped prisoner in 1968. | 1.31 (1.60) | .23 | .05 | Yes |
| *Heath Ledger died from overdosing on prescription drugs.* | 1.48 (1.54) | .18 | .12 | Yes |
| *Spanish princess Maria Teresa was the first member of a royal family to die from COVID-19.* | 1.13 (1.62) | .18 | .09 | Yes |
| An airport in Wisconsin has put on a drive-through lights display. | 0.84 (4.56) | .19 | .21* | Yes |
| *Researchers are making significant progress on curing HIV.* | 1.30 (1.21) | .18 | .24* | Yes |

*Note.* Headlines selected for the final study are in italics.
