# Supplementary material for: Call it a conspiracy: How conspiracy belief predicts recognition of conspiracy theories
Source: PLoS One. 2024 Apr 18;19(4):e0301601. doi: 10.1371/journal.pone.0301601 (PMC11025851; doi:10.1371/journal.pone.0301601)
Supplement: S3 Table — (DOCX) [file pone.0301601.s009.docx]

S3 Table. *Means and standard deviations for belief in each article summary and correlations with demographic variables in Study 1*

|  | M (SD) | Age | Education | Political Orientation | Mainstream Belief | Conspiracy Belief | Response Time | Score |
| --- | --- | --- | --- | --- | --- | --- | --- | --- |
| ConAr1 | 4.25 (1.68) | -.19** | .24** | .37** | .22** | .79** | .23** | -.52** |
| ConAr2 | 3.84 (1.90) | -.19** | .34** | .36** | 0.11 | .72** | .24** | -.59** |
| ConAr3 | 4.41 (1.43) | -.16* | .14* | .25** | .39** | .67** | .19** | -.40** |
| ConAr4 | 4.39 (1.36) | -.15* | .13* | .23** | .32** | .69** | .19** | -.38** |
| ConAr5 | 3.98 (1.74) | -.25** | .32** | .39** | .21** | .84** | .24** | -.62** |
| ConAr6 | 4.36 (1.41) | -.14* | .20** | .13* | .37** | .68** | .16* | -.33** |
| ConAr7 | 4.76 (1.53) | -.10 | .01 | .08 | .29** | .56** | .05 | -.17** |
| ConAr8 | 4.45 (1.49) | -.21** | .16* | .27** | .25** | .71** | .18** | -.30** |
| ConAr9 | 4.67 (1.44) | -.13* | .20** | .14* | .44** | .61** | .10 | -.19** |
| ConAr10 | 3.98 (1.70) | -.17** | .37** | .34** | .26** | .79** | .22** | -.62** |
| MainAr1 | 4.98 (1.37) | .04 | -.04 | -.11 | .56** | .12* | -.05 | .17** |
| MainAr2 | 4.88 (1.38) | -.03 | -.03 | .04 | .63** | .37** | .07 | .06 |
| MainAr3 | 4.95 (1.17) | -.04 | .09 | .09 | .65** | .27** | -.03 | .11 |
| MainAr4 | 4.94 (1.28) | -.02 | -.04 | -.05 | .67** | .23** | -.02 | .11 |
| MainAr5 | 4.68 (1.36) | -.03 | .09 | .09 | .68** | .29** | .08 | -.05 |
| MainAr6 | 5.17 (1.20) | .01 | -.09 | -.05 | .61** | .15* | -.05 | .16* |
| MainAr7 | 4.99 (1.28) | .08 | .00 | .04 | .71** | .18** | .02 | .13* |
| MainAr8 | 4.57 (1.35) | -.12 | .14* | 0.11 | .66** | .41** | .07 | -.09 |
| MainAr9 | 4.77 (1.34) | -.12 | .00 | .11 | .72** | .28** | .06 | .01 |
| MainAr10 | 4.94 (1.28) | -.13* | -.01 | .07 | .65** | .25** | .00 | .09 |
