# Supplementary material for: Call it a conspiracy: How conspiracy belief predicts recognition of conspiracy theories
Source: PLoS One. 2024 Apr 18;19(4):e0301601. doi: 10.1371/journal.pone.0301601 (PMC11025851; doi:10.1371/journal.pone.0301601)
Supplement: S4 Table — (DOCX) [file pone.0301601.s010.docx]

S4 Table. *A full list of headlines for the article summaries in Study 1*

| Label | Headline |
| --- | --- |
| **Conspiracy Articles** |  |
| ConAr1 | COVID-19: Perfect Cover for Mandatory Biometric ID |
| ConAr2 | Coming to a wall or lamppost near you – 5G and fake diseases to cover up its effects |
| ConAr3 | New WikiLeaks Documents Expose Doctoring of Chemical Weapons Report to Justify 2018 US Attack on Syria |
| ConAr4 | New WikiLeaks Documents Expose Doctoring of Chemical Weapons Report to Justify 2018 US Attack on Syria |
| ConAr5 | Psychologist: big tech will use “subliminal methods” to shift 15 million votes on election day |
| ConAr6 | “Undeniable evidence”: Explosive classified docs reveal Afghan war mass deception |
| ConAr7 | Alarming report reveals secretive surveillance state powered by your phone’s location services |
| ConAr8 | New “Out of Shadows” documentary exposes the media and Hollywood for manipulating the masses with lies and propaganda |
| ConAr9 | US Congress cracks down on ABC News for ‘Epstein coverup,’ demands to know who killed the story and why |
| ConAr10 | Did someone murder the wife of a Google whistleblower whose research implicated the tech giant in election meddling? |
| **Mainstream Articles** |  |
| MainAr1 | WHO warning: No evidence that antibody tests can show coronavirus immunity |
| MainAr2 | New Google site shows where people in a community are taking social distancing seriously — and where they're not |
| MainAr3 | FDA approves new drug for patients with metastatic breast cancer |
| MainAr4 | Canada shooting: gunman kills 16 people after rampage in Nova Scotia |
| MainAr5 | Appeals court sides with feds on Jeffrey Epstein deal |
| MainAr6 | World News Updates: Singapore’s Control Slips, as Europe Begins to Ease Coronavirus Limits |
| MainAr7 | East Bay student who made ‘terrorists’ video settles with school district over free speech lawsuit |
| MainAr8 | DOJ review finds material errors in two 2019 surveillance applications |
| MainAr9 | New York 9/11 victim identified 18 years later |
| MainAr10 | Afghan conflict: US and Taliban sign deal to end 18-year war |
