# Supplementary material for: Call it a conspiracy: How conspiracy belief predicts recognition of conspiracy theories
Source: PLoS One. 2024 Apr 18;19(4):e0301601. doi: 10.1371/journal.pone.0301601 (PMC11025851; doi:10.1371/journal.pone.0301601)
Supplement: S5 Table — (DOCX) [file pone.0301601.s011.docx]

S5 Table. *Means and standard deviations for belief in each article summary and correlations with demographic variables in Study 2*

|  | M (SD) | Age | Education | Political Orientation | Mainstream Belief | Conspiracy Belief | Response Time | Score |
| --- | --- | --- | --- | --- | --- | --- | --- | --- |
| ConSt1 | 4.90 (1.34) | -.14* | -.20** | .04 | .35** | .39** | -.04 | .04 |
| ConSt2 | 4.28 (1.79) | -.10 | .07 | .29** | -.09 | .76** | .11 | -.39** |
| ConSt3 | 4.17 (1.79) | -.11 | .07 | .26** | -.13* | .78** | .14* | -.46** |
| ConSt4 | 4.15 (1.63) | -.26** | .10 | .20** | -.06 | .81** | .13* | -.45** |
| ConSt5 | 4.27 (1.66) | -.15* | .01 | .24** | -.05 | .85** | .07 | -.47** |
| ConSt6 | 4.65 (1.43) | -.19** | -.08 | .09 | .23** | .64** | -.03 | -.09 |
| ConSt7 | 4.05 (1.64) | -.27** | .14* | .18** | -.04 | .75** | .07 | -.47** |
| ConSt8 | 3.87 (1.86) | -.19** | .11 | .23** | -.17** | .80** | .14* | -.59** |
| ConSt9 | 4.22 (1.63) | -.20** | .03 | .21** | -.01 | .78** | .12 | -.43** |
| ConSt10 | 4.74 (1.20) | -.17** | -.06 | .00 | .37** | .54** | -.01 | -0.1 |
| MainSt1 | 5.37 (1.30) | .05 | -.22** | -.20** | .75** | .00 | -.04 | .46** |
| MainSt2 | 5.29 (1.33) | .01 | -.09 | -.14* | .70** | .05 | -.06 | .40** |
| MainSt3 | 5.21 (1.31) | -.10 | -.24** | -.24** | .72** | -.10 | -.07 | .43** |
| MainSt4 | 5.37 (1.39) | -.08 | -.13* | -.08 | .78** | .05 | -.04 | .42** |
| MainSt5 | 5.16 (1.39) | -.15* | -.08 | -.08 | .65** | .14* | -.10 | .23** |
| MainSt6 | 5.13 (1.37) | -.04 | -.18** | -.10 | .71** | .01 | -.09 | .28** |
| MainSt7 | 5.26 (1.39) | -.11 | -.12 | -.14* | .74** | -.10 | -.16* | .47** |
| MainSt8 | 5.26 (1.44) | .01 | -.13* | -.01 | .72** | -.09 | .03 | .50** |
| MainSt9 | 5.15 (1.29) | -.15* | -.05 | -.04 | .70** | .18** | -.04 | .27** |
| MainSt10 | 5.21 (1.30) | -.19** | -.04 | -.10 | .73** | .06 | -.06 | .33** |

The means for each item are the average of truth ratings for that item. Truth ratings were on a scale of 1 (Completely False) to 7 (Completely True). “Mainstream Belief” is the average truth ratings for all mainstream statements. “Conspiracy Belief” is the average truth ratings for conspiracy statements. As the ratings for each individual statement are incorporated into the averaged Mainstream Belief and Conspiracy Belief measures, the correlations between mainstream statement belief and the mainstream belief composite variable and the conspiracy statement belief and the conspiracy belief composite variable are essentially item analyses.
