# Supplementary material for: Call it a conspiracy: How conspiracy belief predicts recognition of conspiracy theories
Source: PLoS One. 2024 Apr 18;19(4):e0301601. doi: 10.1371/journal.pone.0301601 (PMC11025851; doi:10.1371/journal.pone.0301601)
Supplement: S6 Table — (DOCX) [file pone.0301601.s012.docx]

S6 Table. *A full list of headlines for the article summaries in Study 2*

| Label | Statement |
| --- | --- |
| **Conspiracy Statements** |  |
| ConSt1 | *Companies that sell smart technology like Google Home and Alexa are collecting information on their customers without their customers’ knowledge and selling that information to third parties.* |
| ConSt2 | *COVID-19 (“the coronavirus”) was created in a lab in China as a bioweapon.* |
| ConSt3 | *Technology companies are suppressing information on the negative health effects of 5G networks.* |
| ConSt4 | *Several members of UK's Parliament were behind the 2005 London bombings in an attempt to increase support for military intervention in the Middle East.* |
| ConSt5 | *Researchers have discovered a cure for cancer, but pharmaceutical companies are suppressing information about it.* |
| ConSt6 | *Jeffery Epstein was assassinated to prevent him from sharing information that would harm powerful politicians.* |
| ConSt7 | *The New England Patriots won against the Jacksonville Jaguars in the 2018 NFL Playoffs because they’d paid off the referees to make calls in their favor.* |
| ConSt8 | *The U.S. government faked the moon landing to gain an advantage in the Cold War over Russia.* |
| ConSt9 | *Princess Diana was assassinated to prevent her from embarrassing the royal family.* |
| ConSt10 | *During the Cold War, the KGB assassinated several scientists that were working on US defense department projects.* |
| **Non-Conspiracy Statements** |  |
| MainSt1 | *Tech companies are investing in new technology that will allow them to automate various tasks including checking out customers at stores and packaging products for shipment.* |
| MainSt2 | *All 50 states in the U.S. require that students are vaccinated before enrolling in public schools, though some exemptions are available for health and religious reasons.* |
| MainSt3 | *The man who drove a car into counter-protesters during the “Unite the Right” rally in Charlottesville, Virginia was charged with first-degree murder and various other offenses.* |
| MainSt4 | *The International Monetary Fund is an international organization that encourages economic cooperation and provides loans to countries in need.* |
| MainSt5 | *Scientists are developing a method to create 3-D printed organs for patients in need.* |
| MainSt6 | *John Lennon was murdered by a man who wanted media attention.* |
| MainSt7 | *The Toronto Raptors won against the Golden State Warriors in the 2019 NBA finals, winning four of the six games in the series.* |
| MainSt8 | *Heath Ledger died from overdosing on prescription drugs.* |
| MainSt9 | *Spanish princess Maria Teresa was the first member of a royal family to die from COVID-19.* |
| MainSt10 | *Researchers are making significant progress on curing HIV.* |
