# Supplementary material for: Call it a conspiracy: How conspiracy belief predicts recognition of conspiracy theories
Source: PLoS One. 2024 Apr 18;19(4):e0301601. doi: 10.1371/journal.pone.0301601 (PMC11025851; doi:10.1371/journal.pone.0301601)
Supplement: S1 Text — A description of the pilot study used to select stimuli. (DOCX) [file pone.0301601.s013.docx]

**S1 Text**

**Pilot**

I conducted a pilot study to identify appropriate stimuli for Studies 1 and 2. The primary goals of this pilot study were to identify headlines and statements that did or did not contain conspiracy theories. I pre-selected 30 headlines (15 intended to be conspiracy headlines and 15 intended to be mainstream headlines) and 30 statements (15 intended to be conspiracy statements and 15 intended to be mainstream statements) for the pilot. I selected conspiracy headlines from websites that appeared on at least five lists of “top conspiracy websites” (e.g., InfoWars, Natural News), and mainstream headlines from several popular mainstream news sites (e.g., CNN, BBC). All headlines were from the past six months. The headlines addressed a variety of topics, and I attempted to match the general topics of the conspiracy and mainstream headlines. I created conspiracy statements based upon famous or contemporary conspiracy theories and created mainstream statements that addressed similar events to the conspiracy statements.

**Method**

***Participants***

One hundred and one workers from Amazon Mechanical Turk completed the pilot survey. Ten participants who showed two or more signs of low-quality responding (Prims & Motyl, 2018), and one participant who indicated that they had misunderstood the instructions were removed, leaving a final sample size of 90 participants. The average age of the sample was 37.43 years (*SD* = 11.12). Twenty-nine participants identified as men, 35 identified as women, and one chose not to answer. . On a scale of -3 (Very Liberal) to 3 (Very Conservative) the sample skewed slightly conservative, with an average political orientation of 0.09 (*SD* = 1.99). Thirty-five participants identified as liberal, 17 identified as moderate, and 38 identified as conservative.

***Measures***

The conspiracy headlines came from several news sites known for their conspiratorial content (InfoWars, ZeroHedge, Natural News, etc.). The headlines were selected because they did not contain references to partisan individuals or policies, and their articles contained elements of conspiracy theories. The mainstream headlines were selected from several of the most trafficked news websites (CNN, Fox, ABC, etc.). These headlines were selected because they did not contain references to partisan individuals or policies, and their articles were missing at least one major element of conspiracy theories (e.g., secrecy).

The statements were written with similar considerations. They did not contain mentions of partisan individuals or policies. Conspiracy statements were based on existing conspiracy theories, and mainstream statements were based on real events. Every headline and statement was accompanied by three bullet points: The first listed who was responsible for the event, the second indicated whether elements of the event were publicized or concealed, and the third indicated the consequences of the event. All headlines and statements are available in Tables 1 and 2. Their accompanying bullet points are available in Appendix A.

Both the motivated reasoning and conspiracy blindness hypotheses predict that people will have trouble recognizing conspiracy theories, so rather than ask participants whether each headline or statement contained a conspiracy theory, I created a check list containing three key features of conspiracy theories: 1) a group of powerful people is working together to accomplish a goal, 2) they are attempting to keep their activity secret, and 3) they are acting at the expense of others (Hawley, 2019, Oliver & Wood, 2014; Uscinski & Parent, 2014). Participants read each headline and statement and indicated every feature they believed was present in that headline or statement. I then tallied the number of features each participant indicated for each headline or statement. A score of 0 indicated that a participant did not see any of the three features in the headline or statement. A score of 3 indicated that the participant saw all of the three features in the headline or statement.

To determine the political leaning of the stimuli (i.e., whether the stimuli primarily appealed to liberals or conservatives), participants reported their belief in the truth of each article and statement on a scale of 1 (Completely false) to 7 (Completely true), and their political orientation on a scale of 1 (Very liberal) to 7 (Very conservative). The belief measure was re-scored to a scale of -3 (Completely false) to 3 (Completely true) and centered on 0 such that negative scores indicated disbelief and positive scores indicated belief. The political orientation measure was rescored to a scale of -3 (Very liberal) to 3 (Very conservative) and centered on 0 such that negative scores indicated a liberal political orientation, and positive scores indicated a conservative political orientation.

Though I did not tell participants that they were identifying conspiracy theories, I was concerned that they might realize the nature of the task and be reluctant to indicate that they’d noticed all three features in the stimuli they believed. As an additional precaution, two independent coders coded each stimulus for the presence or absence of the three features. Interrater reliability was good. The independent coders agreed substantially on whether each stimulus contained all three features of conspiracy theories (Cohen’s κ = .87), and the total number of features in each stimulus (Cohen’s κ = .72; Cohen’s Weighted κ = .90).

***Selection Criteria***

To reduce ambiguous stimuli, I eliminated any stimulus where the independent coders disagreed on whether it contained all three features of conspiracy theories from consideration (n = 3). Then, to ensure that the conspiracy stimuli contained more features of conspiracy theories than the mainstream stimuli, I selected the 10 conspiracy stimuli with the highest percentage of participants reporting that all three features of conspiracy theories were present, and the 10 mainstream stimuli with the lowest percentage of participants reporting that all three features of conspiracy theories were present.

**Results**

Participants identified more conspiracy features in the conspiracy stimuli (headlines: *M* = 2.30, *SD* = 0.71, statements: *M* = 2.32, *SD* = 0.68) than the non-conspiracy stimuli (headlines: *M* =1.46, *SD* = 0.84, statements: *M* = 1.57, *SD* = 0.70) for both the headlines (*t*(89) = 8.97, *p* < .001) and the statements *t*(89) = 9.65, *p* < .001. Belief in the conspiracy stimuli was lower (headlines: *M* = 0.21, *SD* = 1.10, statements: *M* = 0.19, *SD* = 1.11) than for the non-conspiracy stimuli (headlines: *M* = 1.02, *SD* = 0.85, statements: *M* = 1.35, *SD* = 0.91) for both the headlines (*t*(89) = 6.31, *p* < .001) and the statements, *t*(89) = 7.50, *p* < .001.

To ensure that all potential conspiracy stimuli were perceived as having more conspiracy features than all potential mainstream stimuli, I conducted two *t-*tests (one for the headlines, and one for the statements) comparing the “weakest” conspiracy stimuli (i.e., the conspiracy stimuli with the lowest proportion of participants identifying all three features of conspiracy theories in the stimuli) to the “weakest” mainstream stimuli (i.e., the mainstream stimuli with the highest proportion of participants identifying all three features of conspiracy theories in the stimuli). Even when comparing the weakest conspiracy headline with the weakest mainstream headline, participants identified significantly more conspiracy features in the conspiracy headline than in the mainstream headline, *t*(89) = 3.64, *p* < 001. The same was true when comparing the weakest conspiracy and mainstream statements, *t*(89) = 2.50, *p* = .014.

*S1 Table.* Means and standard deviations of belief, the proportion of participants who saw all three conspiracy features, correlations between political orientation and belief, and interrater agreement on the presence of all three conspiracy features

| Statement | Belief  *M* (*SD*) | Proportion | *r* | Agreement |
| --- | --- | --- | --- | --- |
| **Conspiracy Statements** |  |  |  |  |
| *COVID-19: Perfect Cover for Mandatory Biometric ID* | 0.13 (1.97) | .64 | -.38*** | Yes |
| CNN heavily revises article called out for looking suspiciously like Chinese news release | 0.25 (1.69) | .49 | -.36*** | Yes |
| *Coming to a wall or lamppost near you – 5G and fake diseases to cover up its effects* | -0.33 (2.01) | .58 | -.29** | Yes |
| *New WikiLeaks Documents Expose Doctoring of Chemical Weapons Report to Justify 2018 US Attack on Syria* | 0.03 (1.56) | .58 | -.33** | Yes |
| *Psychologist: big tech will use “subliminal methods” to shift 15 million votes on election day* | -0.25 (1.90) | .67 | -.32** | Yes |
| China launches biological warfare agenda: Covertly infiltrates plane with “Trojan Horse” coronavirus carrier | -0.56 (2.03) | .63 | -.43*** | Yes |
| Cover-up: Iran refuses to hand over black box data of plane that crashed over Tehran | 0.79 (1.48) | .45 | -.11 | No |
| *“Undeniable evidence”: Explosive classified docs reveal Afghan war mass deception* | 0.57 (1.63) | .62 | -.13 | Yes |
| The same people who lied about WMDs in Iraq are pushing conflict with Iran | 0.52 (1.52) | .50 | -.07 | Yes |
| *Alarming report reveals secretive surveillance state powered by your phone’s location services* | 0.67 (1.65) | .61 | -.27* | Yes |
| *New “Out of Shadows” documentary exposes the media and Hollywood for manipulating the masses with lies and propaganda* | 0.39 (1.76) | .53 | -.33** | Yes |
| Ads warning about dangers of 5G banned by Great Britain’s advertising “authority” | 0.27 (1.81) | .43 | -.31** | No |
| What Happened on the Planes on September 11, 2001? The 9/11 Cell Phone Calls. The 9/11 Commission “Script” Was Fabricated | -0.29 (1.94) | .41 | -.38*** | Yes |
| *US Congress cracks down on ABC News for ‘Epstein coverup,’ demands to know who killed the story and why* | 0.80 (1.41) | .50 | -.28** | Yes |
| *Did someone murder the wife of a Google whistleblower whose research implicated the tech giant in election meddling?* | -0.31 (1.97) | .55 | -.30** | Yes |
| **Non-Conspiracy Statements** |  |  |  |  |
| *WHO warning: No evidence that antibody tests can show coronavirus immunity* | 1.13 (1.53) | .20 | .21 | Yes |
| Pentagon to extend troop movement freeze to June 30 | 1.27 (1.41) | .22 | -.04 | Yes |
| American voters worry they can’t spot misleading information, poll finds | 1.40 (1.39) | .30 | .16 | Yes |
| Iranian Missile Accidentally Brought Down Ukrainian Jet, Officials Say | 0.90 (1.52) | .28 | -.03 | Yes |
| *New Google site shows where people in a community are taking social distancing seriously — and where they're not* | 1.44 (1.30) | .21 | .07 | Yes |
| *FDA approves new drug for patients with metastatic breast cancer* | 1.38 (1.16) | .18 | -.02 | Yes |
| *Canada shooting: gunman kills 16 people after rampage in Nova Scotia* | 1.32 (1.47) | .20 | .24* | Yes |
| *Appeals court sides with feds on Jeffrey Epstein deal* | 0.65 (1.46) | .20 | -.10 | Yes |
| *World News Updates: Singapore’s Control Slips, as Europe Begins to Ease Coronavirus Limits* | 1.30 (1.31) | .18 | .20 | Yes |
| *East Bay student who made ‘terrorists’ video settles with school district over free speech lawsuit* | 0.63 (1.53) | .20 | -.14 | Yes |
| *DOJ review finds material errors in two 2019 surveillance applications* | 1.07 (1.18) | .21 | .08 | Yes |
| Iran president says Iran responded, will respond to assassination of Soleimani | 1.00 (1.61) | .24 | -.02 | Yes |
| Wikileaks founder Julian Assange denied bail by London court | 1.11 (1.50) | .22 | .01 | Yes |
| *New York 9/11 victim identified 18 years later* | 1.04 (1.59) | .21 | .03 | Yes |
| *Afghan conflict: US and Taliban sign deal to end 18-year war* | 1.12 (1.38) | .19 | -.15 | Yes |

*Note.* Headlines selected for the final study are in italics.

To select the final stimuli for Study 1, I selected the 10 conspiracy headlines with the highest proportion of participants that saw all three features of conspiracy theories in the headline, and the 10 mainstream headlines with the lowest proportion of participants that saw all three features of conspiracy theories in the headline. In the case of a tie, I selected the headline with the highest belief score, to ensure that conspiracy statements had relatively high levels of belief. The headlines of the final stimuli are printed in italics in Table 1.

*S2 Table.* Means and standard deviations of belief, the proportion of participants who saw all three conspiracy features, correlations between political orientation and belief, and interrater agreement on the presence of all three conspiracy features

| Statement | Belief  *M* (*SD*) | Proportion | *r* | Agreement |
| --- | --- | --- | --- | --- |
| **Conspiracy Statements** |  |  |  |  |
| Lobbyists for pharmaceutical companies are pushing state governments to require vaccinations. | 0.40 (1.81) | .52 | -.39*** | Yes |
| The Chinese government is using its influence to force Google to suppress unfavorable information. | 0.92 (1.70) | .52 | -.06 | Yes |
| *Companies that sell smart technology like Google Home and Alexa are collecting information on their customers without their customers’ knowledge and selling that information to third parties.* | 0.77 (1.59) | .62 | -.11 | Yes |
| *COVID-19 (“the coronavirus”) was created in a lab in China as a bioweapon.* | -0.61 (2.16) | .62 | -.51*** | Yes |
| *Technology companies are suppressing information on the negative health effects of 5G networks.* | -0.39 (1.96) | .64 | -.44*** | Yes |
| *Several members of UK's Parliament were behind the 2005 London bombings in an attempt to increase support for military intervention in the Middle East.* | -0.11 (1.98) | .63 | -.34** | Yes |
| Amazon's publicity department has been paying television stations to air scripted statements disguised as news reports. | 0.30 (1.65) | .45 | -.15 | Yes |
| *Researchers have discovered a cure for cancer, but pharmaceutical companies are suppressing information about it.* | -0.15 (2.08) | .59 | -.39*** | Yes |
| *Jeffery Epstein was assassinated to prevent him from sharing information that would harm powerful politicians.* | 0.57 (1.59) | .59 | -.28* | No |
| *The New England Patriots won against the Jacksonville Jaguars in the 2018 NFL Playoffs because they’d paid off the referees to make calls in their favor.* | -0.12 (1.85) | .65 | -.38*** | Yes |
| *The U.S. government faked the moon landing to gain an advantage in the Cold War over Russia.* | -0.37 (2.20) | .54 | -.42*** | Yes |
| Paul McCartney died in the 1960s, and his music label replaced him with a look-alike to avoid losing money | -0.86 (2.02) | .40 | -.41*** | Yes |
| *Princess Diana was assassinated to prevent her from embarrassing the royal family.* | -0.33 (2.01) | .54 | -.36*** | Yes |
| There is a secret weapons testing facility hidden under the Denver Airport. | -0.25 (1.94) | .35 | -.36*** | Yes |
| *During the Cold War, the KGB assassinated several scientists that were working on US defense department projects.* | 0.72 (1.44) | .59 | -.22* | Yes |
| **Non-Conspiracy Statements** |  |  |  |  |
| Several army veterans bombed a federal building in Oklahoma City as retaliation for federal government’s perceived incompetence in several investigations. | 0.69 (1.78) | .29 | -.02 | No |
| The U.S. sent troops to Libya to assist its government in its conflict with several militant groups. | 1.08 (1.20) | .21 | -.18 | Yes |
| *Tech companies are investing in new technology that will allow them to automate various tasks including checking out customers at stores and packaging products for shipment.* | 1.56 (1.17) | .18 | .14 | Yes |
| Some governments are tracking the movement of people who were later diagnosed with COVID-19 to predict which communities will need the most resources. | 1.41 (1.28) | .21 | .12 | Yes |
| *All 50 states in the U.S. require that students are vaccinated before enrolling in public schools, though some exemptions are available for health and religious reasons.* | 1.43 (1.38) | .19 | .11 | Yes |
| *The man who drove a car into counter-protesters during the “Unite the Right” rally in Charlottesville, Virginia was charged with first-degree murder and various other offenses.* | 1.52 (1.40) | .18 | .29** | Yes |
| *The International Monetary Fund is an international organization that encourages economic cooperation and provides loans to countries in need.* | 1.39 (1.37) | .18 | .10 | Yes |
| *Scientists are developing a method to create 3-D printed organs for patients in need.* | 1.22 (1.40) | .15 | -.02 | Yes |
| *John Lennon was murdered by a man who wanted media attention.* | 1.54 (1.54) | .17 | .09 | Yes |
| *The Toronto Raptors won against the Golden State Warriors in the 2019 NBA finals, winning four of the six games in the series.* | 1.44 (1.57) | .18 | .04 | Yes |
| Martin Luther King Jr. was assassinated by an escaped prisoner in 1968. | 1.31 (1.60) | .23 | .05 | Yes |
| *Heath Ledger died from overdosing on prescription drugs.* | 1.48 (1.54) | .18 | .12 | Yes |
| *Spanish princess Maria Teresa was the first member of a royal family to die from COVID-19.* | 1.13 (1.62) | .18 | .09 | Yes |
| An airport in Wisconsin has put on a drive-through lights display. | 0.84 (4.56) | .19 | .21* | Yes |
| *Researchers are making significant progress on curing HIV.* | 1.30 (1.21) | .18 | .24* | Yes |

*Note.* Headlines selected for the final study are in italics.

To select the final stimuli for Study 2, I selected the 10 conspiracy statements with the highest proportion of participants that saw all three features of conspiracy theories in the statement, and the 10 mainstream statements with the lowest proportion of participants that saw all three features of conspiracy theories in the statement. In the case of a tie, I selected the headline with the highest belief score, to ensure that conspiracy statements had relatively high levels of belief. The statements of the final stimuli are printed in italics in Table 2.
