# Supplementary material for: Call it a conspiracy: How conspiracy belief predicts recognition of conspiracy theories
Source: PLoS One. 2024 Apr 18;19(4):e0301601. doi: 10.1371/journal.pone.0301601 (PMC11025851; doi:10.1371/journal.pone.0301601)
Supplement: S2 Text — The full text of the pilot study survey. (DOCX) [file pone.0301601.s014.docx]

S2 Text

hc1 **COVID-19: Perfect cover for mandatory biometric ID**
The article went on to say that... 
- Pharmaceutical and technology companies are pushing for Biometric ID. 
- These companies claim this is to track COVID-19, but they intend to use it for other purposes.
- The Biometric ID could be used to share private health information with these companies without the consent of the patient.

hc2 **CNN heavily revises article called out for looking suspiciously like Chinese news release**
The article went on to say that: 
- CNN wrote the original article in cooperation with the Chinese media. 
- The original article was copied from a Chinese military news release. When readers noticed this, the original article was deleted and is no longer available on CNN's website.
- The original article was propaganda, intended to decrease faith in the United States’ government.

hc3 **Coming to a wall or lamppost near you – 5G and fake diseases to cover up its effects**
The article went on to say that: 
- Governments and technology companies are working to roll out 5G over the next two years.
- These companies claim that 5G is safe, and are suppressing information about its negative health effects. 
- 5G radiation can cause illnesses and damage DNA, cells, and organs.

hc4 **New WikiLeaks Documents Expose Doctoring of Chemical Weapons Report to Justify 2018 US Attack on Syria**
The article went on to say that... 
- The U.S. Government modified official documents.
- The government modified the documents to suppress evidence that Syria's leader was not behind a chemical attack.
- They doctored the report to frame Syria's leader and justify going to war with Syria.

hc5 **Psychologist: big tech will use “subliminal methods” to shift 15 million votes on election day**
The article went on to say that... 
- Search engines and social media websites are intentionally modifying their code to shift voter opinion. 
- These companies have not told anyone. This was uncovered by a reporter. 
- If their plan succeeds, this risks undermining the will of the people.

hc6 **China launches biological warfare agenda: Covertly infiltrates plane with “Trojan Horse” coronavirus carrier**
The article went on to say that... 
- The Chinese government placed someone infected with COVID-19 on a plane to Taiwan.
- This was a covert operation.
- This infected everyone on the plane, and may have accelerated the rate of infection in Taiwan.

hc7 **Cover-up: Iran refuses to hand over black box data of plane that crashed over Tehran**
The article went on to say that... 
- The Iranian government is not allowing investigators to examine evidence of the cause of a plane crash. 
- They are doing this to cover up the true cause of the crash. 
- That some sort of incident occurred on the plane that caused it to crash.

hc8 **"Undeniable Evidence": Explosive Classified Docs Reveal Afghan War Mass Deception**
The article went on to say that... 
- Pentagon leaders were behind the deception. 
- Though they knew that the war was 'unwinnable,' they lied to prolong it. 
- They spent $1 trillion in taxpayer money and prolonged a war that cost many people their lives.

hc9 **The same people who lied about WMDs in Iraq are pushing conflict with Iran**
The article went on to say that... 
- The U.S. government was behind the plot. 
- They claimed that people from Iran were behind terrorist attacks, when there is no evidence that this is the case. 
- This war would lead to many deaths.

hc10 **Alarming report reveals secretive surveillance state powered by your phone’s location services**
The article went on to say that... 
- Tech companies are using location information to track people from one location to another.
- They claim that their location information is anonymized, but it is not. 
- They can sell this information to others without your consent.

hc11 **New “Out of Shadows” documentary exposes the media and Hollywood for manipulating the masses with lies and propaganda**
The article went on to say that...
- The media and Hollywood engage in social engineering to manipulate the public. 
- The media often neglects reporting on the misdeeds of its members. 
- Crimes members of the media commit are swept under the rug.

hc12 **Ads warning about dangers of 5G banned by Great Britain’s advertising “authority”**
The article went on to say that...
- The part of the U.K.'s government that regulates advertisements has banned ads questioning the safety of 5G. 
- By doing this, the U.K. is hiding 5G's negative health effects. 
- An Italian study suggested that exposure to 5G could increase the risk of cancer.

hc13 **What Happened on the Planes on September 11, 2001? The 9/11 Cell Phone Calls. The 9/11 Commission “Script” Was Fabricated**
The article went on to say that... 
- The 9/11 commission wrote the report. 
- The commission claimed they had information from cell phone calls originating from the planes, but the technology to make cell phone calls from airplanes was not available in 2001. 
- This suggests that the 9/11 commission fabricated most of its information and may have lied about the real cause of the 9/11 attacks.

hc14 **US Congress cracks down on ABC News for ‘Epstein coverup,’ demands to know who killed the story and why**
The article went on to say that... 
- ABC network executives were aware of Epstein's crimes many years before they were revealed to the public. 
- These executives told their reporters not to report on the story. 
- If ABC's executives did not bury this story, many of Epstein's victims may have been saved.

hc15 **Did someone murder the wife of a Google whistleblower whose research implicated the tech giant in election meddling?**
The article went on to say that... 
- Google was manipulating search results to influence elections. 
- One of their employees discovered this and tried to share this information. 
- The employee's wife died shortly after. He believes that she was murdered in retaliation.

mh1 **WHO warning: No evidence that antibody tests can show coronavirus immunity**
The article went on to say that... 
- The World Health Organization (WHO) said there is no evidence that contracting and recovering from COVID-19 makes someone immune to the virus. 
- They issued a warning about this information to the public. 
- The WHO hopes this information will prevent further spread of the virus.

mh2 **Pentagon to extend troop movement freeze to June 30**
The article went on to say that...
- The Pentagon has asked troops not to engage in international travel until June 30th. 
- A Pentagon official is quoted, discussing the decision. 
- The Pentagon hopes to slow the spread of COVID-19.

mh3 **American voters worry they can’t spot misleading information, poll finds**
The article went on to say that... 
- A PBS/NPR/Marist poll found that the majority of Americans think that identifying misinformation is difficult. 
- The percentages of each response from the poll is reported. 
- The article expresses concern that misinformation may affect upcoming elections.

mh4 **Iranian Missile Accidentally Brought Down Ukrainian Jet, Officials Say**
The article went on to say that...
- U.S. intelligence and Canadian intelligence suggests that the missile strike was unintentional. 
- The Prime Ministers of Canada and Britain and the President of the United States expressed their belief that it was a mistake to reporters. There will be an investigation, and all information from the investigation will be shared with the public. 
- 176 people died.

mh5 **New Google site shows where people in a community are taking social distancing seriously — and where they're not**
The article went on to say that... 
- Google has launched a website that uses population data to show social distancing in locations like groceries, stores, parks, and homes. 
- The website allows anyone to view its data. 
- They hope this data will be useful for public health departments.

mh6 **FDA approves new drug for patients with metastatic breast cancer**
The article went on to say that... 
- Seattle Genetics created the drug. 
- The director of the FDA made a statement announcing the drug's approval. 
- This provides an additional treatment option for people with this disease.

mh7 **Canada shooting: gunman kills 16 people after rampage in Nova Scotia**
The article went on to say that...
- A single gunman killed 16 people in Nova Scotia. 
- This was done in and around a single house. 
- It was the worst mass shooting in modern Canadian history.

mh8 **Appeals court sides with feds on Jeffrey Epstein deal**
The article went on to say that... 
- The appeals court concluded that the prosecution's actions in the Epstein case did not violate victims' rights. 
- The appeals court wrote a public opinion to explain its decision. 
- A representative of the appeals court further said that, while their decision was consistent with the law, he did not believe that the law was morally sound.

mh9 **World News Updates: Singapore’s Control Slips, as Europe Begins to Ease Coronavirus Limits**
- Singapore's government has loosened restrictions set in place due to COVID-19. 
- Singapore's citizens have taken advantage, going outside and socializing. 
- As a consequence, Singapore is seeing another spike in cases.

mh10 **East Bay student who made ‘terrorists’ video settles with school district over free speech lawsuit**
The article went on to say that...
- A student released a video that some claimed was racist and insensitive. His high school punished him, and he filed a lawsuit claiming that the school district was restricting his free speech.
- After three years, the school district agreed to settle the case. 
- The school district will issue a public apology and pay him and his family $665,000.

mh11 **DOJ review finds material errors in two 2019 surveillance applications**
The article went on to say that... 
- The Department of Justice (DOJ) found errors in two applications for surveillance warrants. 
- The DOJ wrote a report that explained the errors, and steps it was taking to prevent such errors in the future. 
- After a review, the DOJ concluded that, without the errors, their agency would have still reached the same decision for both applications.

mh12 **Iran president says Iran responded, will respond to assassination of Soleimani**
The article went on to say that... 
- Soleimani was killed in a U.S. drone strike. 
- The President announced that the U.S. assassinated Soleimani because he was both directly and indirectly responsible for many deaths.
- Iran's president stated that Soleimani was a national hero.

mh13 **Wikileaks founder Julian Assange denied bail by London court**
The article went on to say that... 
- Assange was charged with 18 criminal counts of hacking. 
- The court and Assange's lawyer both discussed the decision with the media. 
- Assange's lawyer is concerned that his health is too poor for him to attempt to run.

mh14 **New York 9/11 victim identified 18 years later**
The article went on to say that... 
- This is the 1645th victim to be identified after the attack. 
- The press is withholding his name at the request of the family. 
- 40% of those who died remain unidentified.

mh15 **Afghan conflict: US and Taliban sign deal to end 18-year war**
The article went on to say that... 
- The US and NATO allies have agreed to withdraw all of their troops from Afghanistan within 14 months, assuming that the Taliban upholds its end of the deal. 
- The US President made a statement about the agreement at a recent press conference. 
- This would end a war that has killed many soldiers and civilians.

cs1 **Lobbyists for pharmaceutical companies are pushing state governments to require vaccinations.**
- Pharmaceutical companies are motivated to sell vaccines to turn a profit. 
- While they are aware that there are potentially harmful effects of vaccines, they are hiding those effects. 
- These lobbyists are trying to pass laws that require every child to be vaccinated, even against their parents’ wishes.

cs2 **The Chinese government is using its influence to force Google to suppress unfavorable information.**
- The Chinese government has been putting pressure on Google for many years. 
- They have used their power to suppress unfavorable search results, and silence activists. 
- This makes it more difficult for other countries to learn about human rights abuses in China.

cs3 **Companies that sell smart technology like Google Home and Alexa are collecting information on their customers without their customers’ knowledge, and selling that information to third parties.**
- Tech companies have created devices that can record and broadcast information from consumers' homes. 
- They do not inform customers that they are collecting this data, or what they do with this data. 
- They are sharing private information with others without their customers' consent for their own profit.

cs4 **COVID-19 (“the coronavirus”) was created in a lab in China as a bioweapon.**
- The Chinese government funded the creation of the virus. 
- Their researchers are spreading misinformation that the virus emerged naturally. 
- COVID-19 has killed many people and infected many more.

cs5 **Technology companies are suppressing information on the negative health effects of 5G networks.**
- Technology companies have created 5G technology and are building 5G towers all over the world. 
- They claim that 5G is safe and hide information that contradicts them.
- Studies show that 5G can have negative health effects, including increasing a risk of cancer.

cs6 **Several members of UK's Parliament were behind the 2005 London bombings in an attempt to increase support for military intervention in the Middle East.**
- This group acted without the knowledge of the majority of the members of parliament. 
- They framed several Islamic men for the attacks. 
- The attack killed 52 people and injured 700 more.

cs7 **Amazon's publicity department has been paying television stations to air scripted statements disguised as news reports.**
- Amazon's publicity department is responsible for ensuring that the company maintains a good reputation. 
- While Amazon told the station to claim that their source for the stories was a reporter, one station let slip that their source was an Amazon employee. 
- Amazon is using these statements to push its personal political interests.

cs8 **Researchers have discovered a cure for cancer, but pharmaceutical companies are suppressing information about it.**
- If there was a widely available cure for cancer, pharmaceutical companies would lose money because they produce the medication for long-term treatment. 
- Pharmaceutical companies are using their money and influence to prevent news of the cure from becoming widespread. 
- Patients who would otherwise be cured are dying in the absence of a widely available cure.

cs9 **Jeffery Epstein was assassinated to prevent him from sharing information that would harm powerful politicians.**
- Many powerful people were involved in Epstein's sex-trafficking ring. Epstein was in custody and may have revealed their names to the public. 
- These powerful people assassinated Epstein to prevent him from implicating them in the ring. 
- Many people involved in the ring may never be caught and prosecuted and may go on to commit further crimes.

cs10 **The New England Patriots won against the Jacksonville Jaguars in the 2018 NFL Playoffs because they’d paid off the referees to make calls in their favor.**
- The Patriots’ coaches and owner bribed the referees to help them win. 
- The transaction was done in cash to prevent anyone from finding out.
- As a result, the Jaguars lost the NFL Playoffs.

cs11 **The U.S. government faked the moon landing to gain an advantage in the Cold War over Russia.**
- The footage that is currently available was filmed on a television set. There are several clues indicating this, including the flag waving when there is no wind on the moon.
- The U.S. government is suppressing evidence that the moon landing to this day, and attempting to discredit people who have found flaws in the footage.
- Millions of taxpayer dollars have been wasted to maintain this lie.

cs12 **Paul McCartney died in the 1960s, and his music label replaced him with a look-alike to avoid losing money**
- The Beatles were the music label's most popular band at the time. If they lost a member, it would have cost them a huge amount of money. 
- If anyone found out that Paul was replaced, it would be a scandal that would tarnish the label's reputation. 
- The replacement took credit for Paul's accomplishments, and the label continued to make money under false pretenses.

cs13 **Princess Diana was assassinated to prevent her from embarrassing the royal family.**
- The Royal Family has a long-standing reputation to protect. Princess Diana had done something that would have embarrassed the family.  
- The official investigation claimed that Diana was killed in a car accident, but the crash was not an accident.
- Diana was popular among the public, and her death devastated people all over the world.

cs14 **There is a secret weapons testing facility hidden under the Denver Airport.**
- The U.S. government has built a testing facility under the Denver airport. Its location allows them to move people and supplies in large quantities without arousing suspicion.
- The government has consistently denied this facility's existence. 
- This facility creates weapons with massive destructive power.

cs15 **During the Cold War, the KGB assassinated several scientists that were working on US defense department projects.**
- Six scientists that were working on classified projects died under suspicious circumstances in the space of a year.
- Most of the deaths were ruled as suicides or accidents. 
- Several more scientists working on classified projects died under suspicious circumstances in the following years.

ms1 **Several army veterans bombed a federal building in Oklahoma City as retaliation for federal government’s perceived incompetence in several investigations.**
- These veterans drove a truck containing explosives into the building. 
- Both perpetrators were caught on the day of the bombing. 
- 168 people died in the attack and hundreds more were injured.

ms2 **The U.S. sent troops to Libya to assist its government in its conflict with several militant groups.**
- Various countries, including Russia, have sent troops to Libya in recent years.  
- In early 2020, the U.S. announced it was withdrawing all of these troops from Libya. 
- Libya has requested that the U.S. send them troops again to reduce Russia's influence in the country.

ms3 **Tech companies are investing in new technology that will allow them to automate various tasks including checking out customers at stores and packaging products for shipment.**
- Other tasks would include stocking shelves and additional manufacturing processes. 
- Amazon has recently opened a store that uses some of this technology. 
- While this technology would eliminate some jobs, it would create others.

ms4 **Some governments are tracking the movement of people who are diagnosed with COVID-19 to predict which communities will need the most resources.**
- This technology is being used in several countries, and primarily operates using smart phone GPS. 
- The countries using this technology have announced that it is largely successful in slowing the spread of the virus.
- The ability to predict which communities will be hardest hit has allowed these countries to get ahead of the virus, and prepare hospitals for flare-ups in cases.

ms5 **All 50 states in the U.S. require that students are vaccinated before enrolling in public schools, though some exemptions are available for health and religious reasons.**
- State governments have different laws regarding vaccinations. 
- Individual states' laws are available on their official web pages. 
- Vaccination prevents the contraction and spread of serious diseases. However, some people cannot safely receive vaccinations due to compromised immune systems. They rely on others being vaccinated to avoid contracting these diseases.

ms6 **The man who drove a car into counter-protesters during the “Unite the Right” rally in Charlottesville, Virginia was charged with first-degree murder and various other offenses.**
- While the man was attending the rally, he acted alone while driving the car. 
- There were hundreds of witnesses, and his actions were caught on camera. 
- One person was killed, and several more were injured.

ms7 **The International Monetary Fund (IMF) is an international organization that encourages economic cooperation and provides loans to countries in need.**
- The IMF employs people from all over the world. 
- Information about its activities are available on its website. 
- The IMF's mission is ensuring international economic stability to the extent it is possible.

ms8 **Scientists are developing a method to create 3-D printed organs for patients in need.**
- Different labs have created 3D printed organs that can be made of either artificial or organic materials. 
- New breakthroughs are announced frequently, and clinical trials are in progress.
- There is a shortage of organ donors. Advances in artificial organs would help thousands of people across the world.

ms9 **John Lennon was murdered by a man who wanted media attention.**
- The killer's name was Mark David Chapman. 
- He shot John Lennon in front of his apartment building. He then waited at the scene for the police to arrest him. 
- John Lennon was a member of the popular band, the Beatles, which he left several years before his death. People all over the world were saddened by the news.

ms10 **The Toronto Raptors won against the Golden State Warriors in the 2019 NBA finals, winning four of the six games in the series.**
- They won the final game of the series with a score of 114 to 110. 
- The 2019 NBA finals received 20.5 million viewers across the United States and Canada. 
- While the Raptors’ fans were delighted, the Warriors’ fans were disappointed.

ms11 **Martin Luther King Jr. was assassinated by an escaped prisoner in 1968.**
- The assassin was named James Earl Ray. 
- MLK was shot on the balcony of his motel room in view of pedestrians. 
- He later died in the hospital without regaining consciousness.

ms12 **Heath Ledger died from overdosing on prescription drugs.**
- Heath Ledger was an award-winning actor. 
- The overdoes appears to have been accidental. 
- His many fans were saddened by his death.

ms13 **Spanish princess Maria Teresa was the first member of a royal family to die from COVID-19.**
- Princess Maria died on March 26th, 2020. 
- Her memorial service was held the next day and her death was announced on an official website.
- She was dedicated to democracy and social justice in Spain.

ms14 **An airport in Wisconsin has put on a drive-through lights display.**
- The employees put on the display in response to COVID-19. 
- The display was publicized on several travel websites and various news sites. It included lights displays representing destinations like New York City and Las Vegas. 
- The goal of the display was to provide entertainment while maintaining social distancing.

ms15 **Researchers are making significant progress on curing HIV.**
- Researchers all over the world have been working to find a cure for decades. 
- They recently announced that stem cell transplants appear to have cured HIV in two individuals. 
- Formerly an extremely deadly autoimmune disease, we may have a widely available cure for HIV within the next decade.
