# Supplementary material for: Call it a conspiracy: How conspiracy belief predicts recognition of conspiracy theories
Source: PLoS One. 2024 Apr 18;19(4):e0301601. doi: 10.1371/journal.pone.0301601 (PMC11025851; doi:10.1371/journal.pone.0301601)
Supplement: S3 Text — The full text of the Study 1 survey. (DOCX) [file pone.0301601.s015.docx]

S2 Text

In this study, you will be presented with headlines and summaries of various articles. 
 
**First** you will rate how true you believe the statement is. If you are not sure if it is true or false, please select 4 (neither false nor true). 
 
**Second** you will be asked if the article contains a conspiracy theory. Please use your best judgment. 
 
**Third** you will be asked if you would like to explain your answer to the previous question. If you would like to explain your answer, you will be given the opportunity to write an explanation. If you would not like to explain your answer, you will advance to the next headline.

| Page Break |  |
| --- | --- |

You will now see and respond to twenty headlines and article summaries. 


Some of these articles contain conspiracy theories, and some do not. 


Once you are done responding to the headlines and article summaries, you will provide some demographic information, and the survey will end.

**End of Block: Instructions**

**Start of Block: CS1**

**COVID-19: Perfect cover for mandatory biometric ID**

 The article went on to say that... 

 - Pharmaceutical and technology companies are pushing for Biometric ID. 
 - These companies claim this is to track COVID-19, but they intend to use it for other purposes.
 - The Biometric ID could be used to share private health information with these companies without the consent of the patient.

How true is the information in this article on a scale of 1 (Completely False) to 7 (Completely True)? 


Please select 4 (Neither False nor True) if you are not sure.

- 1 - Completely False
- 2
- 3
- 4- Neither False nor True
- 5
- 6
- 7 - Completely True

Does this article contain a conspiracy theory?

- Yes
- No

**End of Block: CS1**

**Start of Block: CS2**

**Coming to a wall or lamppost near you – 5G and fake diseases to cover up its effects**

 The article went on to say that: 

 - Governments and technology companies are working to roll out 5G over the next two years.
 - These companies claim that 5G is safe, and are suppressing information about its negative health effects. 
 - 5G radiation can cause illnesses and damage DNA, cells, and organs.

How true is the information in this article on a scale of 1 (Completely False) to 7 (Completely True)? 


Please select 4 (Neither False nor True) if you are not sure.

- 1 - Completely False
- 2
- 3
- 4- Neither False nor True
- 5
- 6
- 7 - Completely True

Does this article contain a conspiracy theory?

- Yes
- No

**End of Block: CS2**

**Start of Block: CS3**

**New WikiLeaks Documents Expose Doctoring of Chemical Weapons Report to Justify 2018 US Attack on Syria**

 The article went on to say that... 

 - The U.S. Government modified official documents.
 - The government modified the documents to suppress evidence that Syria's leader was not behind a chemical attack.
 - They doctored the report to frame Syria's leader and justify going to war with Syria. 

How true is the information in this article on a scale of 1 (Completely False) to 7 (Completely True)? 


Please select 4 (Neither False nor True) if you are not sure.

- 1 - Completely False
- 2
- 3
- 4- Neither False nor True
- 5
- 6
- 7 - Completely True

Does this article contain a conspiracy theory?

- Yes
- No

**End of Block: CS3**

**Start of Block: CS4**

**New WikiLeaks Documents Expose Doctoring of Chemical Weapons Report to Justify 2018 US Attack on Syria**

 The article went on to say that... 

 - The U.S. Government modified official documents.
 - The government modified the documents to suppress evidence that Syria's leader was not behind a chemical attack.
 - They doctored the report to frame Syria's leader and justify going to war with Syria. 

How true is the information in this article on a scale of 1 (Completely False) to 7 (Completely True)? 


Please select 4 (Neither False nor True) if you are not sure.

- 1 - Completely False
- 2
- 3
- 4- Neither False nor True
- 5
- 6
- 7 - Completely True

Does this article contain a conspiracy theory?

- Yes
- No

**End of Block: CS4**

**Start of Block: CS5**

**Psychologist: big tech will use “subliminal methods” to shift 15 million votes on election day**

 The article went on to say that... 

 - Search engines and social media websites are intentionally modifying their code to shift voter opinion. 
 - These companies have not told anyone. This was uncovered by a reporter. 
 - If their plan succeeds, this risks undermining the will of the people.

How true is the information in this article on a scale of 1 (Completely False) to 7 (Completely True)? 


Please select 4 (Neither False nor True) if you are not sure.

- 1 - Completely False
- 2
- 3
- 4- Neither False nor True
- 5
- 6
- 7 - Completely True

Does this article contain a conspiracy theory?

- Yes
- No

**End of Block: CS5**

**Start of Block: CS6**

**"Undeniable Evidence": Explosive Classified Docs Reveal Afghan War Mass Deception**

 The article went on to say that... 

 - Pentagon leaders were behind the deception. 
 - Though they knew that the war was 'unwinnable,' they lied to prolong it. 
 - They spent $1 trillion in taxpayer money and prolonged a war that cost many people their lives.

How true is the information in this article on a scale of 1 (Completely False) to 7 (Completely True)? 


Please select 4 (Neither False nor True) if you are not sure.

- 1 - Completely False
- 2
- 3
- 4- Neither False nor True
- 5
- 6
- 7 - Completely True

Does this article contain a conspiracy theory?

- Yes
- No

**End of Block: CS6**

**Start of Block: CS7**

**Alarming report reveals secretive surveillance state powered by your phone’s location services**

 The article went on to say that... 

 - Tech companies are using location information to track people from one location to another.
 - They claim that their location information is anonymized, but it is not. 
 - They can sell this information to others without your consent.

How true is the information in this article on a scale of 1 (Completely False) to 7 (Completely True)? 


Please select 4 (Neither False nor True) if you are not sure.

- 1 - Completely False
- 2
- 3
- 4- Neither False nor True
- 5
- 6
- 7 - Completely True

Does this article contain a conspiracy theory?

- Yes
- No

**End of Block: CS7**

**Start of Block: CS8**

**New “Out of Shadows” documentary exposes the media and Hollywood for manipulating the masses with lies and propaganda**
 The article went on to say that...

 - The media and Hollywood engage in social engineering to manipulate the public. 
 - The media often neglects reporting on the misdeeds of its members. 
 - Crimes members of the media commit are swept under the rug.

How true is the information in this article on a scale of 1 (Completely False) to 7 (Completely True)? 


Please select 4 (Neither False nor True) if you are not sure.

- 1 - Completely False
- 2
- 3
- 4- Neither False nor True
- 5
- 6
- 7 - Completely True

Does this article contain a conspiracy theory?

- Yes
- No

**End of Block: CS8**

**Start of Block: CS9**

**US Congress cracks down on ABC News for ‘Epstein coverup,’ demands to know who killed the story and why**

 The article went on to say that... 

 - ABC network executives were aware of Epstein's crimes many years before they were revealed to the public. 
 - These executives told their reporters not to report on the story. 
 - If ABC's executives did not bury this story, many of Epstein's victims may have been saved.

How true is the information in this article on a scale of 1 (Completely False) to 7 (Completely True)? 


Please select 4 (Neither False nor True) if you are not sure.

- 1 - Completely False
- 2
- 3
- 4- Neither False nor True
- 5
- 6
- 7 - Completely True

Does this article contain a conspiracy theory?

- Yes
- No

**End of Block: CS9**

**Start of Block: CS10**

**Did someone murder the wife of a Google whistleblower whose research implicated the tech giant in election meddling?**

 The article went on to say that... 

 - Google was manipulating search results to influence elections. 
 - One of their employees discovered this and tried to share this information. 
 - The employee's wife died shortly after. He believes that she was murdered in retaliation.

How true is the information in this article on a scale of 1 (Completely False) to 7 (Completely True)? 


Please select 4 (Neither False nor True) if you are not sure.

- 1 - Completely False
- 2
- 3
- 4- Neither False nor True
- 5
- 6
- 7 - Completely True

Does this article contain a conspiracy theory?

- Yes
- No

**End of Block: CS10**

**Start of Block: MS1**

**WHO warning: No evidence that antibody tests can show coronavirus immunity**

 The article went on to say that... 

 - The World Health Organization (WHO) said there is no evidence that contracting and recovering from COVID-19 makes someone immune to the virus. 
 - They issued a warning about this information to the public. 
 - The WHO hopes this information will prevent further spread of the virus.

How true is the information in this article on a scale of 1 (Completely False) to 7 (Completely True)? 


Please select 4 (Neither False nor True) if you are not sure.

- 1 - Completely False
- 2
- 3
- 4- Neither False nor True
- 5
- 6
- 7 - Completely True

Does this article contain a conspiracy theory?

- Yes
- No

**End of Block: MS1**

**Start of Block: MS2**

**New Google site shows where people in a community are taking social distancing seriously — and where they're not**


 The article went on to say that... 


 - Google has launched a website that uses population data to show social distancing in locations like groceries, stores, parks, and homes. 
 - The website allows anyone to view its data. 
 - They hope this data will be useful for public health departments.

How true is the information in this article on a scale of 1 (Completely False) to 7 (Completely True)? 


Please select 4 (Neither False nor True) if you are not sure.

- 1 - Completely False
- 2
- 3
- 4- Neither False nor True
- 5
- 6
- 7 - Completely True

Does this article contain a conspiracy theory?

- Yes
- No

**End of Block: MS2**

**Start of Block: MS3**

**FDA approves new drug for patients with metastatic breast cancer**


 The article went on to say that... 


 - Seattle Genetics created the drug. 
 - The director of the FDA made a statement announcing the drug's approval. 
 - This provides an additional treatment option for people with this disease.

How true is the information in this article on a scale of 1 (Completely False) to 7 (Completely True)? 


Please select 4 (Neither False nor True) if you are not sure.

- 1 - Completely False
- 2
- 3
- 4- Neither False nor True
- 5
- 6
- 7 - Completely True

Does this article contain a conspiracy theory?

- Yes
- No

**End of Block: MS3**

**Start of Block: MS4**

**Canada shooting: gunman kills 16 people after rampage in Nova Scotia**


 The article went on to say that...


 - A single gunman killed 16 people in Nova Scotia. 
 - This was done in and around a single house. 
 - It was the worst mass shooting in modern Canadian history.

How true is the information in this article on a scale of 1 (Completely False) to 7 (Completely True)? 


Please select 4 (Neither False nor True) if you are not sure.

- 1 - Completely False
- 2
- 3
- 4- Neither False nor True
- 5
- 6
- 7 - Completely True

Does this article contain a conspiracy theory?

- Yes
- No

**End of Block: MS4**

**Start of Block: MS5**

**Appeals court sides with feds on Jeffrey Epstein deal**


 The article went on to say that... 


 - The appeals court concluded that the prosecution's actions in the Epstein case did not violate victims' rights. 
 - The appeals court wrote a public opinion to explain its decision. 
 - A representative of the appeals court further said that, while their decision was consistent with the law, he did not believe that the law was morally sound.

How true is the information in this article on a scale of 1 (Completely False) to 7 (Completely True)? 


Please select 4 (Neither False nor True) if you are not sure.

- 1 - Completely False
- 2
- 3
- 4- Neither False nor True
- 5
- 6
- 7 - Completely True

Does this article contain a conspiracy theory?

- Yes
- No

**End of Block: MS5**

**Start of Block: MS6**

**World News Updates: Singapore’s Control Slips, as Europe Begins to Ease Coronavirus Limits**


 - Singapore's government has loosened restrictions set in place due to COVID-19. 
 - Singapore's citizens have taken advantage, going outside and socializing. 
 - As a consequence, Singapore is seeing another spike in cases.

How true is the information in this article on a scale of 1 (Completely False) to 7 (Completely True)? 


Please select 4 (Neither False nor True) if you are not sure.

- 1 - Completely False
- 2
- 3
- 4- Neither False nor True
- 5
- 6
- 7 - Completely True

Does this article contain a conspiracy theory?

- Yes
- No

**End of Block: MS6**

**Start of Block: MS7**

**DOJ review finds material errors in two 2019 surveillance applications**


 The article went on to say that... 


 - The Department of Justice (DOJ) found errors in two applications for surveillance warrants. 
 - The DOJ wrote a report that explained the errors, and steps it was taking to prevent such errors in the future. 
 - After a review, the DOJ concluded that, without the errors, their agency would have still reached the same decision for both applications.

How true is the information in this article on a scale of 1 (Completely False) to 7 (Completely True)? 


Please select 4 (Neither False nor True) if you are not sure.

- 1 - Completely False
- 2
- 3
- 4- Neither False nor True
- 5
- 6
- 7 - Completely True

Does this article contain a conspiracy theory?

- Yes
- No

**End of Block: MS7**

**Start of Block: MS8**

**East Bay student who made ‘terrorists’ video settles with school district over free speech lawsuit**


 The article went on to say that...


 - A student released a video that some claimed was racist and insensitive. His high school punished him, and he filed a lawsuit claiming that the school district was restricting his free speech.
 - After three years, the school district agreed to settle the case. 
 - The school district will issue a public apology and pay him and his family $665,000.

How true is the information in this article on a scale of 1 (Completely False) to 7 (Completely True)? 


Please select 4 (Neither False nor True) if you are not sure.

- 1 - Completely False
- 2
- 3
- 4- Neither False nor True
- 5
- 6
- 7 - Completely True

Does this article contain a conspiracy theory?

- Yes
- No

**End of Block: MS8**

**Start of Block: MS9**

**New York 9/11 victim identified 18 years later**


 The article went on to say that... 


 - This is the 1645th victim to be identified after the attack. 
 - The press is withholding his name at the request of the family. 
 - 40% of those who died remain unidentified.

How true is the information in this article on a scale of 1 (Completely False) to 7 (Completely True)? 


Please select 4 (Neither False nor True) if you are not sure.

- 1 - Completely False
- 2
- 3
- 4- Neither False nor True
- 5
- 6
- 7 - Completely True

Does this article contain a conspiracy theory?

- Yes
- No

**End of Block: MS9**

**Start of Block: MS10**

**Afghan conflict: US and Taliban sign deal to end 18-year war**


 The article went on to say that... 


 - The US and NATO allies have agreed to withdraw all of their troops from Afghanistan within 14 months, assuming that the Taliban upholds its end of the deal. 
 - The US President made a statement about the agreement at a recent press conference. 
 - This would end a war that has killed many soldiers and civilians.

How true is the information in this article on a scale of 1 (Completely False) to 7 (Completely True)? 


Please select 4 (Neither False nor True) if you are not sure.

- 1 - Completely False
- 2
- 3
- 4- Neither False nor True
- 5
- 6
- 7 - Completely True

Does this article contain a conspiracy theory?

- Yes
- No

**End of Block: MS10**

**Start of Block: Demographics**

| 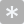 |
| --- |

Please define the term “conspiracy theory” in your own words.

________________________________________________________________

What is your age?

________________________________________________________________

What is your gender?

- Man
- Woman
- Non-binary
- Prefer to self-identify ________________________________________________

What is your political orientation?

- Very liberal
- Liberal
- Slightly liberal
- Moderate
- Slightly conservative
- Conservative
- Very conservative

What is your highest level of education?

- Less than high school
- High school graduate
- Some college
- 2-year degree
- 4-year degree
- Professional degree
- Doctorate

What is your race/ethnicity? (Check all that apply):

- White
- Black or African American
- American Indian or Alaska Native
- Hispanic/Latino
- Asian
- Native Hawaiian or Pacific Islander
- Other

What is your favorite movie?

________________________________________________________________

Is there anything you'd like to add?

________________________________________________________________

________________________________________________________________

________________________________________________________________

________________________________________________________________

________________________________________________________________
