# Supplementary material for: Call it a conspiracy: How conspiracy belief predicts recognition of conspiracy theories
Source: PLoS One. 2024 Apr 18;19(4):e0301601. doi: 10.1371/journal.pone.0301601 (PMC11025851; doi:10.1371/journal.pone.0301601)
Supplement: S4 Text — The full text of the Study 2 survey. (DOCX) [file pone.0301601.s016.docx]

S2 Text

Conspiracy theories are explanations for events or circumstances that claim a group of powerful people is working together to accomplish a goal that comes at the expense of others, while attempting to keep their actions or intentions a secret.

In this study, you will be presented with statements. 
 
**First** you will rate how true you believe the statement is. If you are not sure if it is true or false, please select 4 (neither false nor true). 
 
**Second** you will be asked if the statement contains a conspiracy theory. Please use your best judgment. 
 
**Third** you will be asked if you would like to explain your answer to the previous question. If you would like to explain your answer, you will be given the opportunity to write an explanation. If you would not like to explain your answer, you will advance to the next headline.

| Page Break |  |
| --- | --- |

***Example Statement:*** **Government now forcing tractor manufacturers to build them like smartphones so they can “brick” themselves and become unusable**
- Government officials are working with a prominent tractor manufacturer to change how tractors are made.- They are telling farmers that this is for the sake of keeping tractors updated, but it is really to ensure that they make more money. - Farmers now spend more money every year for less reliable equipment.

|  | Please select all that apply. | | | |
| --- | --- | --- | --- | --- |
|  | A group of people Is trying to accomplish a goal (1) | They are trying to keep their activity secret (2) | They are acting at the expense of others. (3) | None of the 3 apply (4) |
|  |  |  |  |  |

**First, select all that apply:**

 1. A group of people Is trying to accomplish a goal


**This statement mentions that the "government" is doing something to tractor manufacturers. The government is a group of people.**
 
2. These parties are trying to keep their activity secret. (They are trying to hide their methods and/or their goal from the public. This can include things like cover-ups, hoaxes, or simply not sharing information with the public.)
**There is no indication in this statement that the government is hiding its actions.**
 
3. Their actions come at the expense of others. (Their actions hurt or disadvantage many people.)
**By making tractors less reliable, the government is hurting the people that use those tractors.**

 **Second, rate the truth of the statement:**
 Rate how true you believe the statement is on a scale of 1 (Completely False) to 7 (Completely True)
  
If I think that this statement is slightly true: I will select 5

 If I think the statement is completely false: I will select 1 

 I think the statement is equally true and false, or I do not think that I have enough information to know if it is true or false: I will select 4.

**If you believe the statement fits the first and third criteria, and that the statement is slightly true, your response will look like this:**


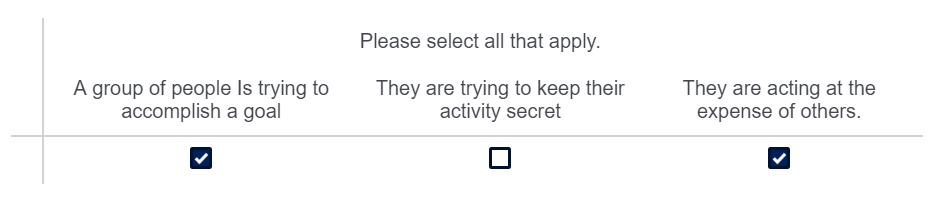


| Page Break |  |
| --- | --- |

You will now see and respond to twenty statements. 


Some of these statements contain conspiracy theories, and some do not. 


Once you are done responding to the statements, you will provide some demographic information, and the survey will end.

**End of Block: Instructions**

**Start of Block: CS1**

Conspiracy theories are explanations for events or circumstances that claim a group of powerful people is working together to accomplish a goal that comes at the expense of others, while attempting to keep their actions or intentions a secret.

**Companies that sell smart technology like Google Home and Alexa are collecting information on their customers without their customers’ knowledge, and selling that information to third parties.**


- Tech companies have created devices that can record and broadcast information from consumers' homes. 
- They do not inform customers that they are collecting this data, or what they do with this data. 
- They are sharing private information with others without their customers' consent for their own profit.

|  | Please select all that apply. | | | |
| --- | --- | --- | --- | --- |
|  | A group of people Is trying to accomplish a goal (1) | They are trying to keep their activity secret (2) | They are acting at the expense of others. (3) | None of the 3 apply (4) |
|  |  |  |  |  |

Does this article contain a conspiracy theory?

- Yes
- No

How true is this statement on a scale of 1 (Completely False) to 7 (Completely True)? 


Please select 4 (Neither False nor True) if you are not sure.

- 1 - Completely False
- 2
- 3
- 4- Neither False nor True
- 5
- 6
- 7 - Completely True

**End of Block: CS1**

**Start of Block: CS2**

Conspiracy theories are explanations for events or circumstances that claim a group of powerful people is working together to accomplish a goal that comes at the expense of others, while attempting to keep their actions or intentions a secret.

**COVID-19 (“the coronavirus”) was created in a lab in China as a bioweapon.**


 - The Chinese government funded the creation of the virus. 
 - Their researchers are spreading misinformation that the virus emerged naturally. 
 - COVID-19 has killed many people and infected many more.

|  | Please select all that apply. | | | |
| --- | --- | --- | --- | --- |
|  | A group of people Is trying to accomplish a goal (1) | They are trying to keep their activity secret (2) | They are acting at the expense of others. (3) | None of the 3 apply (4) |
|  |  |  |  |  |

Does this article contain a conspiracy theory?

- Yes
- No

How true is this statement on a scale of 1 (Completely False) to 7 (Completely True)? 


Please select 4 (Neither False nor True) if you are not sure.

- 1 - Completely False
- 2
- 3
- 4- Neither False nor True
- 5
- 6
- 7 - Completely True

**End of Block: CS2**

**Start of Block: CS3**

Conspiracy theories are explanations for events or circumstances that claim a group of powerful people is working together to accomplish a goal that comes at the expense of others, while attempting to keep their actions or intentions a secret.

**Technology companies are suppressing information on the negative health effects of 5G networks.**


 - Technology companies have created 5G technology and are building 5G towers all over the world. 
 - They claim that 5G is safe and hide information that contradicts them.
 - Studies show that 5G can have negative health effects, including increasing a risk of cancer.

|  | Please select all that apply. | | | |
| --- | --- | --- | --- | --- |
|  | A group of people Is trying to accomplish a goal (1) | They are trying to keep their activity secret (2) | They are acting at the expense of others. (3) | None of the 3 apply (4) |
|  |  |  |  |  |

Does this article contain a conspiracy theory?

- Yes
- No

How true is this statement on a scale of 1 (Completely False) to 7 (Completely True)? 


Please select 4 (Neither False nor True) if you are not sure.

- 1 - Completely False
- 2
- 3
- 4- Neither False nor True
- 5
- 6
- 7 - Completely True

**End of Block: CS3**

**Start of Block: CS4**

Conspiracy theories are explanations for events or circumstances that claim a group of powerful people is working together to accomplish a goal that comes at the expense of others, while attempting to keep their actions or intentions a secret.

**Several members of UK's Parliament were behind the 2005 London bombings in an attempt to increase support for military intervention in the Middle East.**


 - This group acted without the knowledge of the majority of the members of parliament. 
 - They framed several Islamic men for the attacks. 
 - The attack killed 52 people and injured 700 more.

|  | Please select all that apply. | | | |
| --- | --- | --- | --- | --- |
|  | A group of people Is trying to accomplish a goal (1) | They are trying to keep their activity secret (2) | They are acting at the expense of others. (3) | None of the 3 apply (4) |
|  |  |  |  |  |

Does this article contain a conspiracy theory?

- Yes
- No

How true is this statement on a scale of 1 (Completely False) to 7 (Completely True)? 


Please select 4 (Neither False nor True) if you are not sure.

- 1 - Completely False
- 2
- 3
- 4- Neither False nor True
- 5
- 6
- 7 - Completely True

**End of Block: CS4**

**Start of Block: CS5**

Conspiracy theories are explanations for events or circumstances that claim a group of powerful people is working together to accomplish a goal that comes at the expense of others, while attempting to keep their actions or intentions a secret.

**Researchers have discovered a cure for cancer, but pharmaceutical companies are suppressing information about it.**


 - If there was a widely-available cure for cancer, pharmaceutical companies would lose money because they produce the medication for long-term treatment. 
 - Pharmaceutical companies are using their money and influence to prevent news of the cure from becoming widespread. 
 - Patients who would otherwise be cured are dying in the absence of a widely available cure.

|  | Please select all that apply. | | | |
| --- | --- | --- | --- | --- |
|  | A group of people Is trying to accomplish a goal (1) | They are trying to keep their activity secret (2) | They are acting at the expense of others. (3) | None of the 3 apply (4) |
|  |  |  |  |  |

Does this article contain a conspiracy theory?

- Yes
- No

How true is this statement on a scale of 1 (Completely False) to 7 (Completely True)? 


Please select 4 (Neither False nor True) if you are not sure.

- 1 - Completely False
- 2
- 3
- 4- Neither False nor True
- 5
- 6
- 7 - Completely True

**End of Block: CS5**

**Start of Block: CS6**

Conspiracy theories are explanations for events or circumstances that claim a group of powerful people is working together to accomplish a goal that comes at the expense of others, while attempting to keep their actions or intentions a secret.

**Jeffery Epstein was assassinated to prevent him from sharing information that would harm powerful politicians.**


 - Many powerful people were involved in Epstein's sex-trafficking ring. Epstein was in custody and may have revealed their names to the public. 
 - These powerful people assassinated Epstein to prevent him from implicating them in the ring. 
 - Many people involved in the ring may never be caught and prosecuted and may go on to commit further crimes.

|  | Please select all that apply. | | | |
| --- | --- | --- | --- | --- |
|  | A group of people Is trying to accomplish a goal (1) | They are trying to keep their activity secret (2) | They are acting at the expense of others. (3) | None of the 3 apply (4) |
|  |  |  |  |  |

Does this article contain a conspiracy theory?

- Yes
- No

How true is this statement on a scale of 1 (Completely False) to 7 (Completely True)? 


Please select 4 (Neither False nor True) if you are not sure.

- 1 - Completely False
- 2
- 3
- 4- Neither False nor True
- 5
- 6
- 7 - Completely True

**End of Block: CS6**

**Start of Block: CS7**

Conspiracy theories are explanations for events or circumstances that claim a group of powerful people is working together to accomplish a goal that comes at the expense of others, while attempting to keep their actions or intentions a secret.

**The New England Patriots won against the Jacksonville Jaguars in the 2018 NFL Playoffs because they’d paid off the referees to make calls in their favor.**


 - The Patriots’ coaches and owner bribed the referees to help them win. 
 - The transaction was done in cash to prevent anyone from finding out.
 - As a result, the Jaguars lost the NFL Playoffs.

|  | Please select all that apply. | | | |
| --- | --- | --- | --- | --- |
|  | A group of people Is trying to accomplish a goal (1) | They are trying to keep their activity secret (2) | They are acting at the expense of others. (3) | None of the 3 apply (4) |
|  |  |  |  |  |

Does this article contain a conspiracy theory?

- Yes
- No

How true is this statement on a scale of 1 (Completely False) to 7 (Completely True)? 


Please select 4 (Neither False nor True) if you are not sure.

- 1 - Completely False
- 2
- 3
- 4- Neither False nor True
- 5
- 6
- 7 - Completely True

**End of Block: CS7**

**Start of Block: CS8**

Conspiracy theories are explanations for events or circumstances that claim a group of powerful people is working together to accomplish a goal that comes at the expense of others, while attempting to keep their actions or intentions a secret.

**The U.S. government faked the moon landing to gain an advantage in the Cold War over Russia.**


 - The footage that is currently available was filmed on a television set. There are several clues indicating this, including the flag waving when there is no wind on the moon.
 - The U.S. government is suppressing evidence that the moon landing to this day, and attempting to discredit people who have found flaws in the footage.
 - Millions of taxpayer dollars have been wasted to maintain this lie.

|  | Please select all that apply. | | | |
| --- | --- | --- | --- | --- |
|  | A group of people Is trying to accomplish a goal (1) | They are trying to keep their activity secret (2) | They are acting at the expense of others. (3) | None of the 3 apply (4) |
|  |  |  |  |  |

Does this article contain a conspiracy theory?

- Yes
- No

How true is this statement on a scale of 1 (Completely False) to 7 (Completely True)? 


Please select 4 (Neither False nor True) if you are not sure.

- 1 - Completely False
- 2
- 3
- 4- Neither False nor True
- 5
- 6
- 7 - Completely True

**End of Block: CS8**

**Start of Block: CS9**

Conspiracy theories are explanations for events or circumstances that claim a group of powerful people is working together to accomplish a goal that comes at the expense of others, while attempting to keep their actions or intentions a secret.

**Princess Diana was assassinated to prevent her from embarrassing the royal family.**


 - The Royal Family has a long-standing reputation to protect. Princess Diana had done something that would have embarrassed the family.  
 - The official investigation claimed that Diana was killed in a car accident, but the crash was not an accident.
 - Diana was popular among the public, and her death devastated people all over the world.

|  | Please select all that apply. | | | |
| --- | --- | --- | --- | --- |
|  | A group of people Is trying to accomplish a goal (1) | They are trying to keep their activity secret (2) | They are acting at the expense of others. (3) | None of the 3 apply (4) |
|  |  |  |  |  |

Does this article contain a conspiracy theory?

- Yes
- No

How true is this statement on a scale of 1 (Completely False) to 7 (Completely True)? 


Please select 4 (Neither False nor True) if you are not sure.

- 1 - Completely False
- 2
- 3
- 4- Neither False nor True
- 5
- 6
- 7 - Completely True

**End of Block: CS9**

**Start of Block: CS10**

Conspiracy theories are explanations for events or circumstances that claim a group of powerful people is working together to accomplish a goal that comes at the expense of others, while attempting to keep their actions or intentions a secret.

**During the Cold War, the KGB assassinated several scientists that were working on US defense department projects.**


 - Six scientists that were working on classified projects died under suspicious circumstances in the space of a year.
 - Most of the deaths were ruled as suicides or accidents. 
 - Several more scientists working on classified projects died under suspicious circumstances in the following years.

|  | Please select all that apply. | | | |
| --- | --- | --- | --- | --- |
|  | A group of people Is trying to accomplish a goal (1) | They are trying to keep their activity secret (2) | They are acting at the expense of others. (3) | None of the 3 apply (4) |
|  |  |  |  |  |

Does this article contain a conspiracy theory?

- Yes
- No

How true is this statement on a scale of 1 (Completely False) to 7 (Completely True)? 


Please select 4 (Neither False nor True) if you are not sure.

- 1 - Completely False
- 2
- 3
- 4- Neither False nor True
- 5
- 6
- 7 - Completely True

**End of Block: CS10**

**Start of Block: MS1**

Conspiracy theories are explanations for events or circumstances that claim a group of powerful people is working together to accomplish a goal that comes at the expense of others, while attempting to keep their actions or intentions a secret.

**Tech companies are investing in new technology that will allow them to automate various tasks including checking out customers at stores and packaging products for shipment.**


 - Other tasks would include stocking shelves and additional manufacturing processes. 
 - Amazon has recently opened a store that uses some of this technology. 
 - While this technology would eliminate some jobs, it would create others.

|  | Please select all that apply. | | | |
| --- | --- | --- | --- | --- |
|  | A group of people Is trying to accomplish a goal (1) | They are trying to keep their activity secret (2) | They are acting at the expense of others. (3) | None of the 3 apply (4) |
|  |  |  |  |  |

Does this article contain a conspiracy theory?

- Yes
- No

How true is this statement on a scale of 1 (Completely False) to 7 (Completely True)? 


Please select 4 (Neither False nor True) if you are not sure.

- 1 - Completely False
- 2
- 3
- 4- Neither False nor True
- 5
- 6
- 7 - Completely True

**End of Block: MS1**

**Start of Block: MS2**

Conspiracy theories are explanations for events or circumstances that claim a group of powerful people is working together to accomplish a goal that comes at the expense of others, while attempting to keep their actions or intentions a secret.

**All 50 states in the U.S. require that students are vaccinated before enrolling in public schools, though some exemptions are available for health and religious reasons.**


 - State governments have different laws regarding vaccinations. 
 - Individual states' laws are available on their official web pages. 
 - Vaccination prevents the contraction and spread of serious diseases. However, some people cannot safely receive vaccinations due to compromised immune systems. They rely on others being vaccinated to avoid contracting these diseases.

|  | Please select all that apply. | | | |
| --- | --- | --- | --- | --- |
|  | A group of people Is trying to accomplish a goal (1) | They are trying to keep their activity secret (2) | They are acting at the expense of others. (3) | None of the 3 apply (4) |
|  |  |  |  |  |

Does this article contain a conspiracy theory?

- Yes
- No

How true is this statement on a scale of 1 (Completely False) to 7 (Completely True)? 


Please select 4 (Neither False nor True) if you are not sure.

- 1 - Completely False
- 2
- 3
- 4- Neither False nor True
- 5
- 6
- 7 - Completely True

**End of Block: MS2**

**Start of Block: MS3**

Conspiracy theories are explanations for events or circumstances that claim a group of powerful people is working together to accomplish a goal that comes at the expense of others, while attempting to keep their actions or intentions a secret.

**The man who drove a car into counter-protesters during the “Unite the Right” rally in Charlottesville, Virginia was charged with first-degree murder and various other offenses.**


 - While the man was attending the rally, he acted alone while driving the car. 
 - There were hundreds of witnesses, and his actions were caught on camera. 
 - One person was killed, and several more were injured.

|  | Please select all that apply. | | | |
| --- | --- | --- | --- | --- |
|  | A group of people Is trying to accomplish a goal (1) | They are trying to keep their activity secret (2) | They are acting at the expense of others. (3) | None of the 3 apply (4) |
|  |  |  |  |  |

Does this article contain a conspiracy theory?

- Yes
- No

How true is this statement on a scale of 1 (Completely False) to 7 (Completely True)? 


Please select 4 (Neither False nor True) if you are not sure.

- 1 - Completely False
- 2
- 3
- 4- Neither False nor True
- 5
- 6
- 7 - Completely True

**End of Block: MS3**

**Start of Block: MS4**

Conspiracy theories are explanations for events or circumstances that claim a group of powerful people is working together to accomplish a goal that comes at the expense of others, while attempting to keep their actions or intentions a secret.

**The International Monetary Fund (IMF) is an international organization that encourages economic cooperation and provides loans to countries in need.**


 - The IMF employs people from all over the world. 
 - Information about its activities are available on its website. 
 - The IMF's mission is ensuring international economic stability to the extent it is possible.

|  | Please select all that apply. | | | |
| --- | --- | --- | --- | --- |
|  | A group of people Is trying to accomplish a goal (1) | They are trying to keep their activity secret (2) | They are acting at the expense of others. (3) | None of the 3 apply (4) |
|  |  |  |  |  |

Does this article contain a conspiracy theory?

- Yes
- No

How true is this statement on a scale of 1 (Completely False) to 7 (Completely True)? 


Please select 4 (Neither False nor True) if you are not sure.

- 1 - Completely False
- 2
- 3
- 4- Neither False nor True
- 5
- 6
- 7 - Completely True

**End of Block: MS4**

**Start of Block: MS5**

Conspiracy theories are explanations for events or circumstances that claim a group of powerful people is working together to accomplish a goal that comes at the expense of others, while attempting to keep their actions or intentions a secret.

**Scientists are developing a method to create 3-D printed organs for patients in need.**


 - Different labs have created 3D printed organs that can be made of either artificial or organic materials. 
 - New breakthroughs are announced frequently, and clinical trials are in progress.
 - There is a shortage of organ donors. Advances in artificial organs would help thousands of people across the world.

|  | Please select all that apply. | | |
| --- | --- | --- | --- |
|  | A group of people Is trying to accomplish a goal | They are trying to keep their activity secret | They are acting at the expense of others. |
|  |  |  |  |

Does this article contain a conspiracy theory?

- Yes
- No

How true is this statement on a scale of 1 (Completely False) to 7 (Completely True)? 


Please select 4 (Neither False nor True) if you are not sure.

- 1 - Completely False
- 2
- 3
- 4- Neither False nor True
- 5
- 6
- 7 - Completely True

**End of Block: MS5**

**Start of Block: MS6**

Conspiracy theories are explanations for events or circumstances that claim a group of powerful people is working together to accomplish a goal that comes at the expense of others, while attempting to keep their actions or intentions a secret.

**John Lennon was murdered by a man who wanted media attention.**


 - The killer's name was Mark David Chapman. 
 - He shot John Lennon in front of his apartment building. He then waited at the scene for the police to arrest him. 
 - John Lennon was a member of the popular band, the Beatles, which he left several years before his death. People all over the world were saddened by the news.

|  | Please select all that apply. | | | |
| --- | --- | --- | --- | --- |
|  | A group of people Is trying to accomplish a goal (1) | They are trying to keep their activity secret (2) | They are acting at the expense of others. (3) | None of the 3 apply (4) |
|  |  |  |  |  |

Does this article contain a conspiracy theory?

- Yes
- No

How true is this statement on a scale of 1 (Completely False) to 7 (Completely True)? 


Please select 4 (Neither False nor True) if you are not sure.

- 1 - Completely False
- 2
- 3
- 4- Neither False nor True
- 5
- 6
- 7 - Completely True

**End of Block: MS6**

**Start of Block: MS7**

Conspiracy theories are explanations for events or circumstances that claim a group of powerful people is working together to accomplish a goal that comes at the expense of others, while attempting to keep their actions or intentions a secret.

**The Toronto Raptors won against the Golden State Warriors in the 2019 NBA finals, winning four of the six games in the series.**


 - They won the final game of the series with a score of 114 to 110. 
 - The 2019 NBA finals received 20.5 million viewers across the United States and Canada. 
 - While the Raptors’ fans were delighted, the Warriors’ fans were disappointed.

|  | Please select all that apply. | | | |
| --- | --- | --- | --- | --- |
|  | A group of people Is trying to accomplish a goal (1) | They are trying to keep their activity secret (2) | They are acting at the expense of others. (3) | None of the 3 apply (4) |
|  |  |  |  |  |

Does this article contain a conspiracy theory?

- Yes
- No

How true is this statement on a scale of 1 (Completely False) to 7 (Completely True)? 


Please select 4 (Neither False nor True) if you are not sure.

- 1 - Completely False
- 2
- 3
- 4- Neither False nor True
- 5
- 6
- 7 - Completely True

**End of Block: MS7**

**Start of Block: MS8**

Conspiracy theories are explanations for events or circumstances that claim a group of powerful people is working together to accomplish a goal that comes at the expense of others, while attempting to keep their actions or intentions a secret.

**Heath Ledger died from overdosing on prescription drugs.**


 - Heath Ledger was an award-winning actor. 
 - The overdoes appears to have been accidental. 
 - His many fans were saddened by his death.

|  | Please select all that apply. | | | |
| --- | --- | --- | --- | --- |
|  | A group of people Is trying to accomplish a goal (1) | They are trying to keep their activity secret (2) | They are acting at the expense of others. (3) | None of the 3 apply (4) |
|  |  |  |  |  |

Does this article contain a conspiracy theory?

- Yes
- No

How true is this statement on a scale of 1 (Completely False) to 7 (Completely True)? 


Please select 4 (Neither False nor True) if you are not sure.

- 1 - Completely False
- 2
- 3
- 4- Neither False nor True
- 5
- 6
- 7 - Completely True

**End of Block: MS8**

**Start of Block: MS9**

Conspiracy theories are explanations for events or circumstances that claim a group of powerful people is working together to accomplish a goal that comes at the expense of others, while attempting to keep their actions or intentions a secret.

**Spanish princess Maria Teresa was the first member of a royal family to die from COVID-19.**


 - Princess Maria died on March 26th, 2020. 
 - Her memorial service was held the next day and her death was announced on an official website.
 - She was dedicated to democracy and social justice in Spain.

|  | Please select all that apply. | | | |
| --- | --- | --- | --- | --- |
|  | A group of people Is trying to accomplish a goal (1) | They are trying to keep their activity secret (2) | They are acting at the expense of others. (3) | None of the 3 apply (4) |
|  |  |  |  |  |

Does this article contain a conspiracy theory?

- Yes
- No

How true is this statement on a scale of 1 (Completely False) to 7 (Completely True)? 


Please select 4 (Neither False nor True) if you are not sure.

- 1 - Completely False
- 2
- 3
- 4- Neither False nor True
- 5
- 6
- 7 - Completely True

**End of Block: MS9**

**Start of Block: MS10**

Conspiracy theories are explanations for events or circumstances that claim a group of powerful people is working together to accomplish a goal that comes at the expense of others, while attempting to keep their actions or intentions a secret.

**Researchers are making significant progress on curing HIV.**


 - Researchers all over the world have been working to find a cure for decades. 
 - They recently announced that stem cell transplants appear to have cured HIV in two individuals. 
 - Formerly an extremely deadly autoimmune disease, we may have a widely available cure for HIV within the next decade.

|  | Please select all that apply. | | | |
| --- | --- | --- | --- | --- |
|  | A group of people Is trying to accomplish a goal (1) | They are trying to keep their activity secret (2) | They are acting at the expense of others. (3) | None of the 3 apply (4) |
|  |  |  |  |  |

Does this article contain a conspiracy theory?

- Yes
- No

How true is this statement on a scale of 1 (Completely False) to 7 (Completely True)? 


Please select 4 (Neither False nor True) if you are not sure.

- 1 - Completely False
- 2
- 3
- 4- Neither False nor True
- 5
- 6
- 7 - Completely True

**End of Block: MS10**

**Start of Block: Demographics**

| 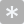 |
| --- |

What is your age?

________________________________________________________________

What is your gender?

- Man
- Woman
- Non-binary
- Prefer to self-identify ________________________________________________

What is your political orientation?

- Very liberal
- Liberal
- Slightly liberal
- Moderate
- Slightly conservative
- Conservative
- Very conservative

What is your highest level of education?

- Less than high school
- High school graduate
- Some college
- 2-year degree
- 4-year degree
- Professional degree
- Doctorate

What is your race/ethnicity? (Check all that apply):

- White
- Black or African American
- American Indian or Alaska Native
- Hispanic/Latino
- Asian
- Native Hawaiian or Pacific Islander
- Other

What is your favorite movie?

________________________________________________________________

Is there anything you'd like to add?

________________________________________________________________

________________________________________________________________

________________________________________________________________

________________________________________________________________

________________________________________________________________
